# The histone chaperone HIRA promotes the induction of host innate immune defences in response to HSV-1 infection

**DOI:** 10.1371/journal.ppat.1007667

**Published:** 2019-03-22

**Authors:** Steven McFarlane, Anne Orr, Ashley P. E. Roberts, Kristen L. Conn, Victor Iliev, Colin Loney, Ana da Silva Filipe, Katherine Smollett, Quan Gu, Neil Robertson, Peter D. Adams, Taranjit Singh Rai, Chris Boutell

**Affiliations:** 1 MRC-University of Glasgow Centre for Virus Research (CVR), Garscube Campus, Glasgow, Scotland, United Kingdom; 2 Department of Veterinary Microbiology, Western College of Veterinary Medicine, University of Saskatchewan, Saskatoon, Saskatoon, CA; 3 Li Ka Shing Institute of Virology, University of Alberta, Edmonton, Alberta, CA; 4 Beatson Institute for Cancer Research, Glasgow, Scotland, United Kingdom; 5 Sanford Burnham Prebys Medical Discovery Institute, San Diego, CA, United States of America; 6 Northern Ireland Centre for Stratified Medicine, Biomedical Sciences Research Institute, Ulster University, Londonderry, United Kingdom; University of Wisconsin-Madison, UNITED STATES

## Abstract

Host innate immune defences play a critical role in restricting the intracellular propagation and pathogenesis of invading viral pathogens. Here we show that the histone H3.3 chaperone HIRA (histone cell cycle regulator) associates with promyelocytic leukaemia nuclear bodies (PML-NBs) to stimulate the induction of innate immune defences against herpes simplex virus 1 (HSV-1) infection. Following the activation of innate immune signalling, HIRA localized at PML-NBs in a Janus-Associated Kinase (JAK), Cyclin Dependent Kinase (CDK), and Sp100-dependent manner. RNA-seq analysis revealed that HIRA promoted the transcriptional upregulation of a broad repertoire of host genes that regulate innate immunity to HSV-1 infection, including those involved in MHC-I antigen presentation, cytokine signalling, and interferon stimulated gene (ISG) expression. ChIP-seq analysis revealed that PML, the principle scaffolding protein of PML-NBs, was required for the enrichment of HIRA onto ISGs, identifying a role for PML in the HIRA-dependent regulation of innate immunity to virus infection. Our data identifies independent roles for HIRA in the intrinsic silencing of viral gene expression and the induction of innate immune defences to restrict the initiation and propagation of HSV-1 infection, respectively. These intracellular host defences are antagonized by the HSV-1 ubiquitin ligase ICP0, which disrupts the stable recruitment of HIRA to infecting viral genomes and PML-NBs at spatiotemporally distinct phases of infection. Our study highlights the importance of histone chaperones to regulate multiple phases of intracellular immunity to virus infection, findings that are likely to be highly pertinent in the cellular restriction of many clinically important viral pathogens.

## Author summary

Host innate immune defences play critical roles in the cellular restriction of invading viral pathogens and the stimulation of adaptive immune responses. A key component in the regulation of this arm of host immunity is the rapid induction of cytokine signalling and the expression of interferon stimulated gene products (ISGs), which confer a refractory antiviral state to limit virus propagation and pathogenesis. While the signal transduction cascades that activate innate immune defences are well established, little is known about the cellular host factors that expedite the expression of this broad repertoire of antiviral host genes in response to pathogen invasion. Here we show that HIRA, a histone H3.3 chaperone, associates with PML-NBs to stimulate the induction of innate immune defences in response to HSV-1 infection. Our study highlights the importance of histone chaperones in the coordinated regulation of multiple phases of host immunity in response to pathogen invasion and identifies a key role for HIRA in the induction of innate immunity to virus infection.

## Introduction

The intrinsic, innate, and adaptive arms of host immunity cooperatively supress the replication and spread of invading viral pathogens. Conferred by constitutively expressed host factors, intrinsic immunity (also known as intrinsic antiviral resistance and cell autonomous immunity) restricts virus replication from the outset of infection [1–4]. By way of contrast, activation of Pattern Recognition Receptors (PRRs) by microbial specific Pathogen-Associated Molecular Patterns (PAMPs) leads to the induction of innate immune defences and the coordinated upregulation of a broad repertoire of host antiviral genes, principally cytokines (including interferons; IFNs) and interferon stimulated gene (ISG) products [3–6]. This induced immune response confers an enhanced antiviral state to limit virus spread and prime adaptive immune responses. Accordingly, many viruses have evolved counter measures to antagonize intrinsic and innate immune defences to promote the efficient onset of replication, propagation, and transmission to new hosts.

A component step in the activation of intrinsic and innate immune defences during herpesvirus infection is the rapid recruitment of host factors to infecting viral genomes, which enter the nucleus as naked linear dsDNA molecules [1, 3, 7, 8]. Recent microscopy and biochemical evidence has shown that the sequential recruitment of host factors to nuclear infecting viral genomes plays an important role in the spatial and temporal (spatiotemporal) regulation of intrinsic and innate immune defences, through the assembly of viral genomes into chromatin that can be epigenetically silenced and the activation of innate PRRs, respectively [9–19]. Notably, many of these host factors have been shown to either reside or transiently associate with promyelocytic leukaemia nuclear bodies (PML-NBs, also known as ND10; [9, 11, 17, 20–26]), which rapidly associate with and entrap infecting viral DNA (vDNA) as a component of the intrinsic antiviral immune response [9, 12, 17, 19, 27–31]. Saturation of intrinsic host defences under high genome loads leads to the induction of innate immune defences in a PML- and IFI16 (interferon gamma inducible protein 16)-dependent manner, which establishes an antiviral state to restrict virus propagation and spread [9, 13, 32–36]. Importantly, intrinsic host defences can inhibit the onset of HSV-1 lytic replication in restrictive cell types independently of the induction of innate immune defences [9, 37].

The importance of PML-NBs in the regulation of intracellular immunity is highlighted by the fact that many DNA and RNA viruses have evolved independent strategies to disrupt these dynamic nuclear sub-domains during infection [1, 3, 38]. One of the first proteins expressed during Herpes Simplex Virus 1 (HSV-1) infection is ICP0, a viral RING-finger ubiquitin ligase with SUMO-Targeting Ubiquitin Ligase (STUbL) properties [23, 39, 40]. ICP0 mediates the degradation and dispersal of host factors, including PML and IFI16 [9, 23, 36, 41–45], away from infecting viral genomes to disable intrinsic and innate immune defences activated in response to infection [1, 3, 46]. Viral mutants that do not express functionally active ICP0 are highly susceptible to intrinsic silencing under low multiplicities of infection (MOI) and are hypersensitive to interferon (IFN) treatment [47–52]. The use of such mutants has been critical for the characterization of many aspects relating to the antiviral roles of PML-NBs in the regulation of intracellular immunity during virus infection.

Recent studies have shown that the HIRA (histone cell cycle regulator) histone H3.3 chaperone complex, composed of HIRA, Ubinuclein 1 (UBN1), and Calcineurin-binding protein 1 (CABIN1), relocalizes to PML-NBs in response to replication-defective herpesvirus infection [17, 18]. This host response has been linked to the intrinsic epigenetic silencing of viral gene expression through the HIRA-dependent deposition of variant histone H3.3 onto viral genomes at PML-NBs in restrictive cell types [17, 18, 53]. This finding is consistent with the replication-independent assembly of HSV-1 genomes into chromatin containing H3.3 shortly after nuclear entry (< 1 hour post-infection (hpi); [16, 18, 19, 53, 54]). Importantly, infections with replication defective herpesviruses prematurely activate PPRs to trigger innate immune defences leading to the secretion of cytokines. This host defence is naturally antagonized by ICP0 during wild-type (WT) HSV-1 infection under physiological infection conditions [9, 33, 36, 55–59]. Notably, exogenous cytokine stimulation (IFN-β and IFN-γ) can induce HIRA localization to PML-NBs independently of infection [17], supporting a role for HIRA at PML-NBs as a component of the innate immune response. Exogenous IFN stimulation has been shown to enrich HIRA at gene bodies across the human genome, including a subset of ISGs [17, 60, 61]. However, the extent to which HIRA influences the activation or regulation of innate immunity during HSV-1 infection remains to be established. We therefore sought to investigate the spatiotemporal kinetics of HIRA localization to infecting HSV-1 genomes and PML-NBs in the sequential regulation of intrinsic and innate immune defences under productive lytic infection conditions pertinent to a clinical setting.

We show that the recruitment of HIRA to infecting HSV-1 genomes, as a component of the intrinsic antiviral immune response, occurs with kinetics that are temporally distinct to those of PML, the principle scaffolding protein of PML-NBs [62, 63]. Under productive infection conditions that promote the activation of innate immune defences, HIRA localized to PML-NBs in cells proximal to a developing infection in a Janus-Associated Kinase (JAK) dependent manner, identifying a role for virus-induced cytokine signalling in the recruitment of HIRA to PML-NBs. RNA-seq analysis revealed that HIRA was required for the efficient induction of cellular genes involved in the regulation of innate immunity, including MHC-I antigen presentation, cytokine signalling, and the expression of a broad repertoire of ISGs. ChIP-seq analysis revealed that PML promoted HIRA enrichment at ISG bodies, identifying a role for PML in the HIRA-dependent regulation of innate immune defences. Depletion of HIRA inhibited ISG expression, which led to a relief in the cellular restriction of ΔICP0 HSV-1 propagation. Our research uncovers distinct kinetics in the spatiotemporal recruitment of HIRA to infecting viral genomes and PML-NBs during different phases of HSV-1 infection and identifies dual roles for HIRA in the sequential regulation of intrinsic and innate immunity to herpesvirus infection. Our data demonstrates the importance of histone chaperones to regulate multiple phases of intracellular host immunity, findings likely to be highly pertinent in the cellular restriction of many clinically important viral pathogens.

## Results

### Virus induced cytokine signalling stimulates the recruitment of HIRA to PML-NBs

**Fig 1:**
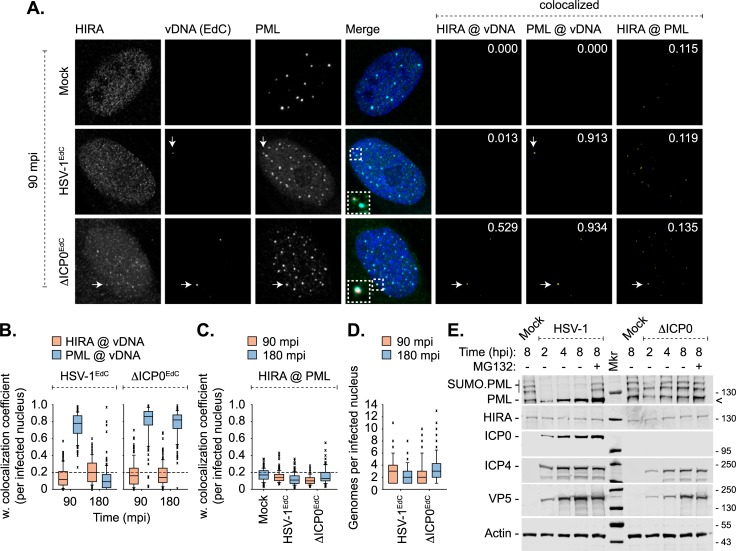
Nuclear entry of HSV-1 genomes is not sufficient to induce HIRA localization at PML-NBs. HFt cells were mock infected or infected with 3 PFU/cell of HSV-1^EdC^ or ΔICP0^EdC^. Cells were fixed and permeabilized at 90 or 180 minutes post-infection (mpi; post-addition of virus). Infecting EdC labelled viral DNA (vDNA) was detected by click chemistry [9]. HIRA and PML were detected by indirect immunofluorescence. (A) Sub-nuclear localization of HIRA (green) and PML (cyan) with respect to infecting HSV-1^EdC^ or ΔICP0^EdC^ vDNA (red, white arrows) at 90 mpi. Insets show magnified regions of interest (dashed boxes). Cut mask (yellow) highlights regions of colocalization between cellular proteins of interest and vDNA or cellular proteins of interest only (as indicated). Weighted colocalization coefficients shown. Nuclei were stained with DAPI (blue). (B, C) Quantitation of cellular protein recruitment to infecting vDNA (B) or HIRA colocalization with PML in mock, HSV-1^EdC^, or ΔICP0^EdC^ infected nuclei (C) at 90 or 180 mpi (as indicated). (D) Genome copy number per infected nuclei at 90 or 180 mpi. Boxes: 25^th^ to 75^th^ percentile range; black line: median weighted (w.) colocalization coefficient; whiskers: 5^th^ to 95^th^ percentile range; crosses: outliers; dashed line: coincident threshold (weighted colocalization coefficients < 0.2). n ≥ 100 nuclei per sample population derived from a minimum of three independent infections. (E) HFt cells were mock, WT, or ΔICP0 HSV-1 infected with 10 PFU/cell in the absence or presence of the proteasome inhibitor MG132 (5 μM; -/+, as indicated). Whole cell lysates were collected at the indicated times for western blot analysis to monitor PML and HIRA stability during HSV-1 infection and the accumulation of viral immediate early (IE; ICP0, ICP4) and late (L; VP5) proteins. Actin is shown as a loading control. Molecular mass markers are shown (Mkr), < denotes the detection of a non-specific background band.

Recent studies have reported that HIRA accumulates at PML-NBs in response to infection with replication-defective herpesviruses (HSV-1 and HCMV) [17, 18]. This host response was linked to the intrinsic epigenetic restriction of viral gene expression at PML-NBs [17, 18]. However, the stable localization of HIRA at PML-NBs was only observed at comparatively late times post-infection (2–6 days; [17, 18]), which is atypical of intrinsic PML-NB host factors that silence viral gene expression from the outset of nuclear infection [9, 19, 27–31]. We hypothesized that this delayed recruitment may be indicative of innate immune defences activated in response to replication-defective herpesvirus infection. We therefore examined the kinetics of HIRA recruitment to infecting WT or ICP0-null mutant (ΔICP0) HSV-1 genomes pre-labelled with EdC (5-Ethynyl-2’-deoxycytidine; HSV-1^EdC^ and ΔICP0^EdC^, respectively) and PML-NBs (Fig 1). Antibody validation experiments confirmed the specificity of the HIRA monoclonal antibody used for indirect immunofluorescence and western blotting in this study (S1 Fig). Consistent with recent studies [9, 10, 64], infecting HSV-1^EdC^ genomes were readily detectible by click chemistry following virion release into the nucleus and to be stably associated with PML-NBs by 90 minutes post-infection (mpi), prior to their disruption and release by ICP0 by 180 mpi (Fig 1A and 1B; [9, 10]). At these times, HIRA did not stably localize to either vDNA or PML-NBs (Fig 1A–1C). It is possible that low levels of ICP0 expression were sufficient to disperse HIRA away from vDNA prior to the disruption of PML-NBs [9]. To test this, HIRA localization was evaluated during ΔICP0^EdC^ HSV-1 infection. While HIRA was observed to localize with vDNA and PML-NBs on rare occasions (Fig 1A, ΔICP0^EdC^), quantitation revealed that the frequency of this colocalization was below coincident threshold levels (weighted colocalization frequency < 0.2, dotted line; Mock) at both 90 and 180 mpi (Fig 1B and 1C). In contrast, PML remained stably colocalized with ΔICP0^EdC^ vDNA (median weighted colocalization frequency > 0.8, Fig 1B; [9]). Genome counts demonstrated that HSV-1^EdC^ and ΔICP0^EdC^ infected nuclei contained equivalent input genome loads (median average 2–3 genomes per nuclei, Fig 1D). As bio-orthogonal nucleic acid labelling is not compatible with live-cell kinetic studies, we cannot rule out the possibility of highly transient or low antigen abundance HIRA interactions with either vDNA or PML-NBs. We conclude that HIRA does not stably colocalize with input vDNA or PML-NBs under infection conditions in which key intrinsic PML-NB restriction factors (PML, Sp100, Daxx, and ATRX; [9]) rapidly entrap and silence vDNA following its nuclear entry.

As ICP0 induces the proteasome-dependent degradation of host restriction factors [12, 45, 46], we next examined the stability of HIRA over the course of WT or ΔICP0 HSV-1 infection. In contrast to PML, which is a well-characterized substrate of ICP0 [23, 39–44, 65], western blot analysis of infected whole cell lysates revealed that HIRA protein levels remained stable throughout infection (Fig 1E). We conclude that HIRA is not a substrate for ICP0 mediated degradation.

**Fig 2:**
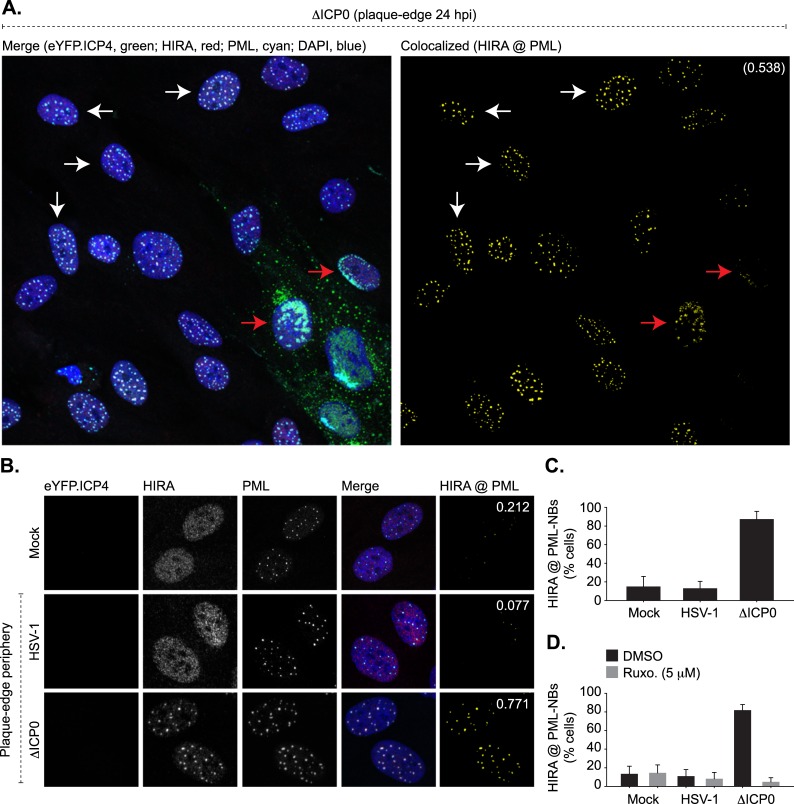
HSV-1 induced activation of innate immune signalling promotes HIRA localization at PML-NBs in a JAK-dependent manner. HFt cells were mock infected or infected with 0.001 or 2 PFU/cell of WT or ΔICP0 HSV-1 recombinant viruses expressing eYFP.ICP4, respectively, to enable plaque-formation to occur. Infected cell monolayers were fixed and permeabilized at 24 hpi, and the nuclear localization of HIRA (red) and PML (cyan) were evaluated by indirect immunofluorescence. Nuclei were stained with DAPI (blue). (A) Wide-field plaque-edge image showing the nuclear localization of HIRA and PML in cells in close proximity to a developing ΔICP0 plaque-edge (eYFP.ICP4, green). Corresponding wide-field images for mock and HSV-1 infected cells are shown in S2 Fig. Cut mask (yellow) highlights regions of colocalization between HIRA and PML. Weighted colocalization coefficient is shown. Arrows highlight examples of cells that are either positive (red) or negative (white) for eYFP.ICP4 expression. (B) Nuclear localization of HIRA and PML in mock or eYFP.ICP4 negative cells at the periphery of developing WT or ΔICP0 plaque-edges (equivalent to white arrows in A). (C) Quantitation of the population of eYFP.ICP4 negative cells showing HIRA colocalization at PML-NBs (as shown in A, B, and S2 Fig). n ≥ 500 cells per sample population derived from four independent infections. Means and standard deviations (SD) shown. (D) Quantitation of the population of eYFP.ICP4 negative cells positive for HIRA colocalization at PML-NBs in mock, WT, or ΔICP0 HSV-1 infected cells treated with DMSO or the JAK inhibitor Ruxolitinib (Ruxo; 5 μM). n ≥ 500 cells per sample population derived from four independent infections. Means and SD shown. Wide-field images for ΔICP0 HSV-1 infected and DMSO or Ruxo treated cell monolayers are shown in S4 Fig.

As nuclear infection in and of itself was not sufficient to induce the stable recruitment of HIRA to vDNA or PML-NBs by 180 mpi, we next evaluated HIRA localization at PML-NBs under infection conditions that enabled the induction of innate immune defences [9]. To this end, cell monolayers were infected at a low-multiplicity of infection to enable the formation of viral plaques by 24 hpi (Fig 2, S2 and S3 Figs). Under these infection conditions, cells on the periphery of developing plaques would be exposed to innate immune signals derived from the infected cells within the body of the adjacent plaque. Cells were infected with recombinant viruses that express eYFP.ICP4 (the major immediate early viral transcription factor) to enable visual identification of productively infected cells, where eYFP-ICP4 accumulates in viral replication compartments prior to cytoplasmic export (Fig 2A, S2 and S3 Figs; red arrows). Within mock- or WT HSV-1 infected cell monolayers, HIRA remained dispersed throughout the nucleus with little to no localization at PML-NBs, either within uninfected (no detectable ICP4 expression) or infected (detectable ICP4 expression) cells (Fig 2B and 2C, S2 Fig). In contrast, in cell monolayers infected with ΔICP0 HSV-1 HIRA stably localized at PML-NBs in both ICP4 positive and negative cells (Fig 2A, S3 Fig; red and white arrows, respectively). Up to 80% of ICP4 negative cells within ΔICP0 HSV-1 infected monolayers had stable HIRA localization at PML-NBs at 24 hpi (Fig 2B and 2C), with HIRA detectable at the majority of individual PML-NBs within any given nucleus (Fig 2B). These data are consistent with the cytokine-mediated induction of signal-transduction cascades stimulating the recruitment of HIRA to PML-NBs in cells proximal to a developing ΔICP0 HSV-1 infection; a host response antagonized by ICP0 during WT HSV-1 infection through the degradation and dispersal of innate immune regulators, including the vDNA PRR IFI16, from infecting viral genomes which suppresses the induction of IFN expression [9, 36, 57, 58]. To test the role of JAK-STAT signalling in the relocalization of HIRA to PML-NBs we examined HIRA localization in ΔICP0 HSV-1 infected cell monolayers treated with the JAK inhibitor Ruxolitinib (Ruxo) [9, 66, 67]. Ruxolitinib treatment effectively abolished the stable localization of HIRA at PML-NBs in ΔICP0 HSV-1 infected cell monolayers (Fig 2D, S4 Fig). We conclude that virus-induced cytokine signalling stimulates the recruitment of HIRA to PML-NBs in response to an emerging HSV-1 infection in a JAK-dependent manner. These data are consistent with findings reported by Rai *et al*., which show exogenous IFN stimulation to promote the stable localization of HIRA at PML-NBs independently of virus infection [17]. Taken together, these data suggest a role for HIRA at PML-NBs as a component of the host innate immune response to prime cells for imminent infection [32, 33, 68].

### IFN induced HIRA localization at PML-NBs is cell-type, CDK, and Sp100-dependent

**Fig 3:**
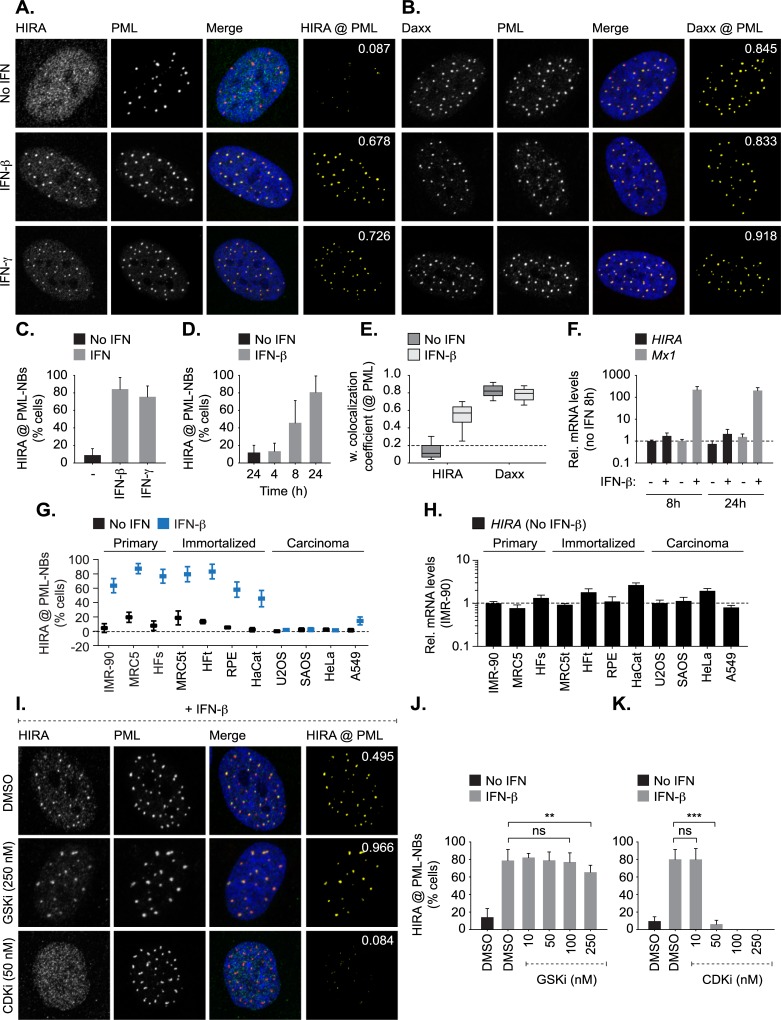
HIRA localizes at PML-NBs in response to exogenous IFN stimulation in a cell type and CDK-dependent manner. Primary, immortalized, or carcinoma cells were mock treated or stimulated with IFN-β or IFN-γ (100 IU/ml) as indicated. Cell monolayers were fixed and permeabilized at 24 h post-stimulation (unless stated otherwise). (A, B) Nuclear localization of HIRA or Daxx (green, as indicated) with respect to PML (red) in mock, IFN-β, or IFN-γ treated (as indicated) HFt cells. Nuclei were stained with DAPI (blue). Cut mask (yellow) highlights regions of colocalization between HIRA or Daxx and PML (as indicated). Weighted colocalization coefficients shown. (C) Quantitation of the population of HFt cells positive for HIRA localization at PML-NBs in mock or IFN treated HFt cells (as shown in A). n ≥ 200 cells per sample population derived from four independent treatments. Means and SD shown. (D) Quantitation of the population of cells positive for HIRA localization at PML-NBs in mock or IFN-β treated HFt cells at 4, 8, or 24 h post-treatment. n ≥ 300 cells per sample population derived from four independent treatments. Means and SD shown. (E) Quantitation of HIRA or Daxx colocalization with PML in HFt cells (as shown in A, B). Boxes: 25^th^ to 75^th^ percentile range; black line: median weighted (w.) colocalization coefficient; whiskers: 5^th^ to 95^th^ percentile range; dashed line: coincident threshold (weighted colocalization coefficients < 0.2). n ≥ 250 cells per sample population derived from a minimum of four independent treatments. (F) qRT-PCR quantitation of *HIRA* or *Mx1* mRNA levels in mock or IFN-β treated HFt cells at 8 or 24 h post-treatment. Values normalized to 8 h no IFN. n = 3, means and SD shown. (G) Quantitation of the population of cells positive for HIRA localization at PML-NBs in mock or IFN-β treated primary (IMR-90, MRC5, HFs), immortalized (MRC5t, HFt, RPE, HaCat), or carcinoma (U2OS, SAOS, HeLa, A549) cells. n ≥ 300 cells per sample population derived from four independent treatments. Means and SD shown. Representative images for each cell line -/+ IFN-β treatment are shown in S5 Fig. (H) qRT-PCR quantitation of *HIRA* mRNA levels in unstimulated primary, immortalized, or carcinoma cells (as in G). Values normalized to the levels of *HIRA* mRNA in primary IMR-90 cells. Mean (RQ) and SD (RQ min/max) shown. (I) Nuclear localization of HIRA (green) and PML (red) in HFt cells pre-treated with DMSO (carrier control), CHIR-99021 HCl (GSK-3α/β inhibitor; GSKi, 250 nM), or flavopiridol (CDK inhibitor; CDKi, 50 nM), for 1 h prior to IFN-β stimulation in the presence of drug for 24 h. Cut mask (yellow) highlights regions of colocalization between HIRA and PML. Weighted colocalization coefficients shown. (J, K) Quantitation of the population of cells positive for HIRA localization at PML-NBs following treatment with DMSO, GSKi, or CDKi over a range of concentrations (as indicated) prior to stimulation (or not) with IFN-β in the presence of drug. n≥ 400 cells per sample population derived from a minimum of four independent treatments. Means and SD shown. ** *P* < 0.01, *** *P* < 0.001, ns (not significant); Mann-Whitney *U*-test.

To investigate the potential roles of HIRA localization at PML-NBs as a component of the innate immune response, we first corroborated and extended HIRA localization studies to PML-NBs following exogenous IFN stimulation (Fig 3; [17]). Stimulation of hTERT immortalized diploid foreskin fibroblast (HFt) cells with type-I (IFN-β) or type-II (IFN-γ) IFN efficiently induced the recruitment of HIRA to PML-NBs in 80% of cells by 24 h post-treatment (Fig 3A and 3C–3E). These data confirm that HIRA recruitment to PML-NBs can occur independently of cellular senescence and virus infection [17, 69–71]. The recruitment of HIRA to PML-NBs did not alter the sub-cellular localization of the resident PML-NB histone H3.3 chaperone Daxx (death domain associated protein 6, [62, 72, 73]; Fig 3B and 3E). The accumulation of HIRA at PML-NBs following IFN treatment also occurred independently of the induction of *HIRA* transcription (Fig 3F), confirming that *HIRA* is not an ISG [17]. HIRA recruitment to PML-NBs following IFN-β stimulation occurred in a range of primary (IMR-90, MRC5, and HFs) and immortalized (MRC5t, HFt, RPE, and HaCat) fibroblast and epithelial cell types (Fig 3G). In contrast, IFN stimulation of carcinoma cells (U2OS, SAOS, HeLa, and A549) failed to induce the recruitment of HIRA to PML-NBs (Fig 3G, S5 Fig; [17]), even though these cell types expressed similar levels of HIRA mRNA and protein within the nucleus (Fig 3H, S5 Fig.). Collectively, these data corroborate and extend findings by Rai *et al*. [17], showing that HIRA localization at PML-NBs in response to IFN stimulation occurs in a cell-type dependent manner and is independent of the induction of cellular senescence.

Phosphoproteomic studies have shown HIRA to be extensively phosphorylated (https://www.phosphosite.org/proteinAction?id=1279&showAllSites=true), with *in vitro* studies supporting a role for cyclin-dependent kinase 2 (CDK2) and glycogen synthase kinase 3β (GSK-3β) phosphorylation in the regulation of HIRA H3.3 chaperone activity and sub-cellular localization [70, 74]. HIRA phosphorylation by GSK-3β has been linked to its localization at PML-NBs during cellular senescence [70]. We therefore examined the potential role of phosphorylation in the recruitment of HIRA to PML-NBs following IFN-β stimulation. Inhibition of GSK-3β only had a modest effect on HIRA localization at PML-NBs in response to cytokine stimulation (250 nM GSKi, CHIR-99021; IC_50_ ≤ 10 nM; Fig 3I and 3J), at doses sufficient to inhibit GSK-3β dependent cell cycle arrest (S6 Fig; [75]). In contrast, HIRA remained nuclear diffuse following IFN-β stimulation in cells treated with Flavopiridol (≥ 50 nM; IC_50_ ≤ 200 nM; Fig 3I and 3K), a broad-spectrum CDK inhibitor (CDKi). Notably, Flavopiridol is also known to inhibit RNA polymerase II transcript elongation through the inhibition of P-TEFb (CDK9/cyclin T1) [76–78]. We conclude that CDKs, either directly or indirectly, influence HIRA recruitment to PML-NBs following IFN-β stimulation. Further studies are warranted to determine the role of CDK phosphorylation in the regulation of HIRA recruitment to PML-NBs in response to cytokine signalling.

**Fig 4:**
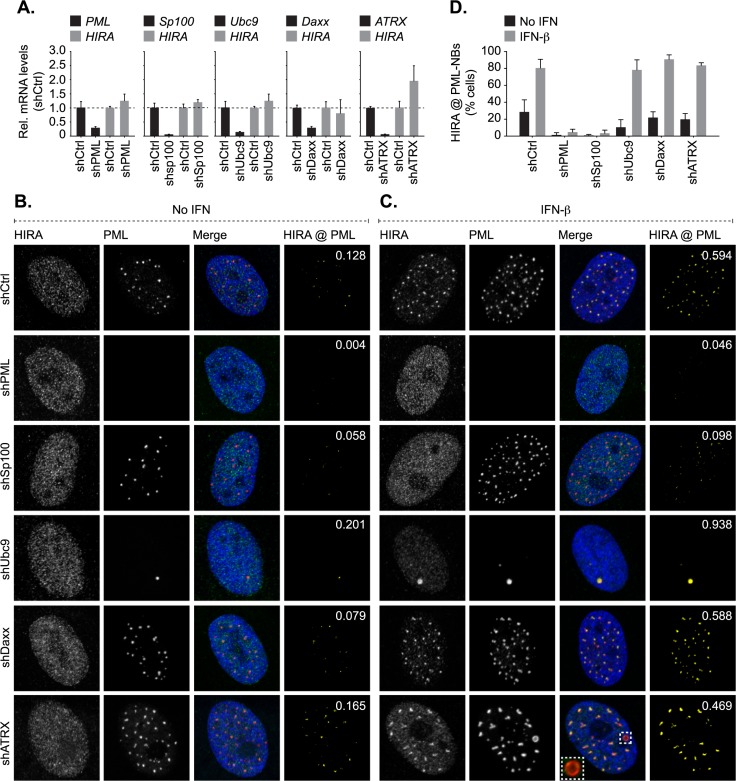
HIRA localization at PML-NBs in response to cytokine stimulation occurs in a PML and Sp100 dependent manner. HFt cells were stably transduced to express individual non-targeting control (shCtrl) or PML-, Sp100-, Ubc9-, Daxx-, or ATRX-targeting (shPML, shSp100, shUbc9, shDaxx, or shATRX, respectively) shRNAs. (A) qRT-PCR quantitation of *PML*, *Sp100*, *Ubc9*, *Daxx*, *ATRX*, or *HIRA* mRNA levels in stably depleted HFt cell lines (as indicated). Means (RQ) and SD (RQ min/max) shown and expressed relative to shCtrl cells. (B, C) Nuclear localization of HIRA (green) and PML (red) in shCtrl, shPML, shSp100, shUbc9, shDaxx, or shATRX cells mock treated or stimulated with IFN-β (100 IU/ml) for 24 h. Nuclei were stained with DAPI (blue). Inset shows magnified region of interest (dashed boxes). Cut mask (yellow) highlights regions of colocalization between HIRA and PML. Weighted colocalization coefficients shown. (D) Quantitation of the population of HFt cells positive for HIRA localization at PML-NBs (as in B). n ≥ 300 cells per sample population derived from four independent treatments. Means and SD shown.

We next evaluated HIRA localization at PML-NBs in cells depleted of individual PML-NB constituent proteins to test their respective roles in the recruitment of HIRA following IFN stimulation (Fig 4, S7 Fig). HFt cells were stably transduced with lentiviral vectors expressing non-targeting control (shCtrl) or PML-, Sp100-, Ubc9-, Daxx-, or ATRX-targeting shRNAs (shPML, shSp100, shUbc9, shDaxx, and shATRX, respectively; as described [23, 27–29, 79]). Depletion of core PML-NB constituent proteins did not reduce *HIRA* mRNA expression or alter its nuclear distribution relative to that of control cells (Fig 4A and 4B). Following IFN-β stimulation, HIRA localized at PML-NBs in cells depleted of Daxx and ATRX (α-thalassemia mental retardation X-linked protein; Fig 4C and 4D), demonstrating that HIRA recruitment to PML-NBs can occur independently of this resident histone H3.3 chaperone complex [62, 72, 73]. In contrast, HIRA localization at PML-NBs was abrogated in the absence of PML, the main scaffolding protein of PML-NBs [62, 63], or Sp100 (Fig 4C and 4D, S7A Fig). In the absence of either PML or Sp100, HIRA did not substantially accumulate into nuclear foci following IFN stimulation. As Sp100 is not required for PML-NB formation [62], these data demonstrate that HIRA accumulates at pre-existing PML-NBs, as opposed to nucleating the accumulation of PML-NB proteins from the nucleoplasm at HIRA foci. As covalent and non-covalent interactions with SUMO play an integral role in mediating many protein-protein interactions associated with PML-NBs [23, 63, 80], we examined the role of SUMOylation in the localization of HIRA at PML-NBs. Disruption of the SUMO pathway through the depletion of Ubc9, the sole E2 SUMO conjugating enzyme, is known to inhibit PML and Sp100 SUMO-modification leading to the formation of enlarged PML nuclear aggregates (one to two foci per nucleus) that contain Sp100 [23]. Following IFN stimulation, HIRA was readily observed to localize at PML nuclear aggregates in Ubc9 depleted cells (Fig 4C and 4D). As Sp100 has been shown to interact with PML in a SUMO-modification independent manner and not to influence PML SUMO-modification directly [28, 81], these data suggest that HIRA recruitment to PML-NBs occurs in a manner that is dependent on Sp100 but independent of *de novo* covalent SUMO-modification. Correspondingly, the molecular mass of endogenous HIRA remained unaltered in HFt cells treated with IFN-β (S7C Fig), indicating that HIRA itself is not extensively SUMOylated to promote its localization at PML-NBs in response to cytokine stimulation. Collectively, these data suggest that Sp100 plays an important role in mediating the recruitment of HIRA to PML-NBs in response to cytokine signalling. However, in contrast to immunoprecipitation studies that have shown HIRA to interact with PML [82], we were unable to detect an interaction between HIRA and Sp100 following IFN stimulation (S7D Fig). Thus, the localization and retention of HIRA at PML-NBs in response to cytokine stimulation is likely to be multifactorial, indicative of the complex network of interactions associated with PML-NBs which rapidly alter in number, size, and distribution is response to cytokine stimulation [38, 83]. Notably, a minor population of IFN stimulated cells could be observed to contain enlarged ‘donut shaped’ foci that contained both PML and Sp100 but not HIRA (Fig 4C, S7B Fig; inserts). While the significance of this minor population of enlarged foci remains to be determined, these data highlight that additional factors are likely to influence HIRA recruitment to PML-NBs in response to IFN stimulation. We conclude that PML and Sp100 are both required to mediate the recruitment and retention of HIRA at PML-NBs in the majority of foci. However, further studies are warranted to identify the precise network of protein-protein interactions required to facilitate the stable localization of HIRA at PML-NBs following IFN stimulation.

### HIRA enhances cytokine signalling and ISG expression during HSV-1 infection

**Fig 5:**
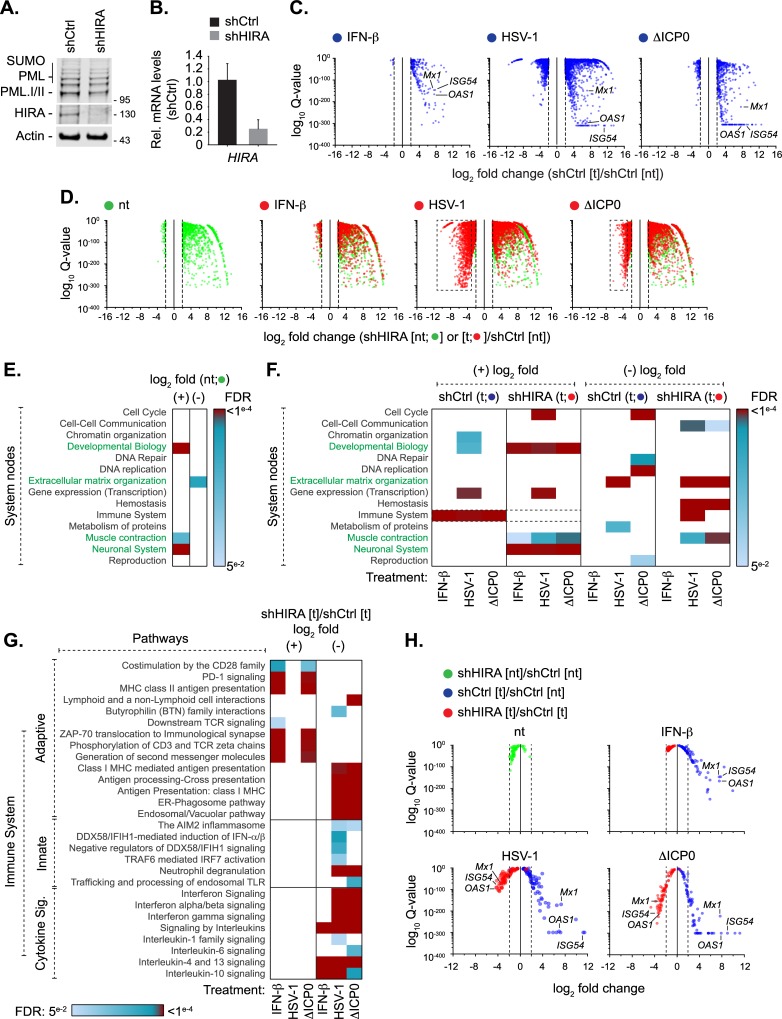
HIRA mediates the induction of host innate immune defences in response to HSV-1 infection. HFt cells were stably transduced to express shRNAs targeting HIRA (shHIRA) or non-targeting control (shCtrl). Cells were either mock treated (no treatment, [nt]) or stimulated with IFN-β (100 IU/ml) or infected with HSV-1 WT or ΔICP0 (MOI 1 PFU/cell) for 17 h (treated, [t]) prior to RNA extraction for RNA-seq analysis. (A) Western blot analysis of the expression levels of PML or HIRA in whole cell lysates derived from shCtrl or shHIRA cells. Actin is shown as a loading control. Molecular mass markers are shown. (B) qRT-PCR quantitation of *HIRA* mRNA levels in shCtrl or shHIRA cells. n = 3, means and SD shown and expressed relative to shCtrl cells. (C) Scatter plots showing high confidence changes in cellular transcript abundance in shCtrl cells treated with IFN-β or infected with HSV-1 WT or ΔICP0 (as indicated; shCtrl [t]) expressed relative to their abundance in non-treated shCtrl cells (shCtrl [nt]; FDR Q-values ≤ 0.0001, ≥ log2 fold change, vertical dotted lines). (D) Scatter plots showing high confidence changes in cellular transcript abundance in shHIRA cells either not treated (green circles, [nt]) or treated with IFN-β or infected with HSV-1 WT or ΔICP0 (as indicated; red circles [t]) expressed relative to their levels in non-treated shCtrl cells (shCtrl [nt]; FDR Q-value ≤ 0.0001, ≥ log2 fold change). Dotted boxes highlight clusters of significantly downregulated genes in HIRA depleted cells infected with WT or ΔICP0 HSV-1. (E) Reactome (https://reactome.org/) pathway analysis of high confidence transcriptome changes (described in D) identified between shHIRA and shCtrl cells (green circles in D). Heat map shows reactome FDR (corrected over-representation *P* value) for pathway nodes enriched in high-confidence mapped entities (Listed in S2 Table). FDR < 0.05 to ≤ 0.0001 (light blue to dark red, significant change); FDR > 0.05 (white, no significant change). Pathway nodes enriched in HIRA depleted cells are highlighted in green text. (F) Heat map (as above) showing pathway analysis for enriched pathway nodes identified in shCtrl (blue circles in C; S1 Table) or shHIRA (red circles in D; S3 Table) cells treated with IFN-β or infected with HSV-1 WT or ΔICP0 (as indicated) relative to non-treated control cells (shCtrl [nt]). (G) Heat map (as above) showing an expanded immune system node highlighting enriched pathways identified in shHIRA cells treated with IFN-β or infected with HSV-1 WT or ΔICP0 (as indicated; shHIRA [t]) relative to similarly treated or infected shCtrl cells (shCtrl [t]; S4 and S5 Tables). (H) Scatter plots showing relative transcript levels (FDR Q-values < 0.05) of core human ISGs [84] in untreated (nt) or treated (t) shCtrl or shHIRA cells (as indicated; S6 Table). Vertical dotted lines highlight log2 fold change cut off for reference. Position of selected ISGs (*Mx1*, *ISG54*, and *OAS1*) highlighted throughout for reference.

In order to investigate the role of HIRA in the regulation of intracellular immunity during virus infection, we conducted RNA-seq analysis in HSV-1 infected cells depleted of HIRA (Fig 5). HFt cells were stably transduced with lentiviral vectors expressing non-targeting control or HIRA-targeting shRNAs (shCtrl and shHIRA, respectively). qPCR and western blotting confirmed HIRA depletion without influencing PML expression levels (Fig 5A and 5B, S1 Fig). Cells were mock treated (no treatment, nt), or either treated (t) with IFN-β (100 IU/ml) or infected with WT or ΔICP0 HSV-1 (MOI 1 PFU/cell) for 17 h prior to RNA extraction for next-generation sequencing (NGS). High confidence reads (FDR Q-value ≤ 0.0001, ≥ log2 fold change) were used for gene expression and gene ontology (GO) analysis (Fig 5C–5G). As expected, treatment with IFN-β or infection with HSV-1 (WT or ΔICP0) induced a strong transcriptional response in comparison to non-treated control cells, with an enrichment in upregulated genes involved in immune system regulation (Fig 5C and 5F [shCtrl (+) log2 fold change]; S1 Table). Depletion of HIRA alone led to a significant shift in gene expression (Fig 5D), with an enrichment in upregulated genes involved in developmental biology, muscle contraction, and neuronal systems (Fig 5E (+) log2 fold change; S2 Table), and a downregulation of genes involved in extracellular matrix organization (Fig 5E (-) log2 fold change). Relative to control cells, HIRA depleted cells did not significantly upregulate the expression of genes involved in immune system regulation in response to IFN treatment or infection with HSV-1 (WT or ΔICP0; Fig 5F shCtrl to shHIRA (+) log2 fold change, dotted box; S1 and S3 Tables). These data suggest that HIRA directly or indirectly promotes the transcriptional upregulation of genes involved in the immune response to HSV-1 infection or IFN-β stimulation. An expanded analysis of the immune system node revealed that infection (WT or ΔICP0 HSV-1) of HIRA depleted cells resulted in a significant downregulation in the expression of genes involved in MHC class I antigen presentation, neutrophil degranulation, and interferon signalling relative to infected control cells (Fig 5G [(-) log2 fold change]; S4 and S5 Tables). These data indicate that HIRA plays a critical role in the transcriptional regulation of a wide variety of genes involved in the immune response to virus infection. Correspondingly, transcriptome profiling of ISG expression revealed that this group of immuno-regulatory genes was downregulated in HIRA depleted cells treated with IFN or infected with HSV-1 (WT or ΔICP0) relative to treated or infected control cells (Fig 5H, S6 Table) [84]. Notably, HSV-1 infection of HIRA-depleted cells resulted in a higher degree of ISG downregulation relative to that observed in HIRA-depleted cells treated with IFN-β (Fig 5H; red dots), highlighting that the nature of immune stimulus can differentially influence the transcriptional induction of ISGs. Taken together, we conclude that HIRA plays important roles in the transcriptional upregulation of a broad repertoire of genes involved in the innate immune response to HSV-1 infection or IFN stimulation.

### PML enhances the deposition of HIRA onto ISGs in response to IFN-β stimulation

**Fig 6:**
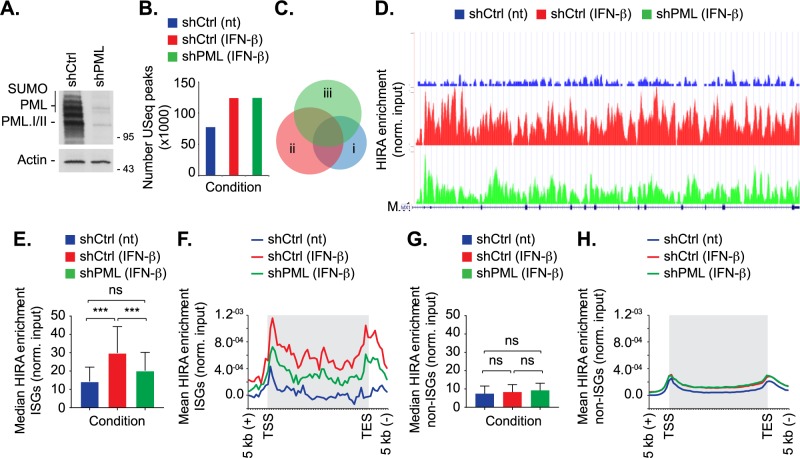
PML enhances HIRA binding to ISGs following IFN-β stimulation. Primary fibroblast (IMR-90) cells were stably transduced to express non-targeting control (shCtrl) or PML-targeting (shPML) shRNAs. Cells were either mock treated (no treatment, nt) or stimulated with IFN-β (2000 IU/ml) for 24 h prior to native ChIP-seq using a panel of HIRA mAbs [69, 74]. (A) Western blot analysis of the expression levels of PML in whole cell lysates derived from untreated shCtrl or shPML cells. Actin is shown as a loading control. Molecular mass markers are indicated. (B) Bar chart showing the number of unique sequencing (USeq) peaks identified following HIRA ChIP-seq for each treatment condition (as indicated; S7 Table). (C) Venn diagram showing the intersection in bp for each condition (i, shCtrl (nt); ii, shCtrl + IFN-β; iii, shPML + IFN-β). (D) Representative UCSC plot of the ISG *Mx1* showing relative HIRA binding under each condition (as indicated). The Y-axis represents library normalised read count. For the gene track, introns are shown as horizontal lines and exons as vertical boxes. (E) Bar graph showing median gene body HIRA enrichment at 49 core ISGs under each condition (as indicated; S7 Table). 95% confidence interval shown. *** *P* < 0.001; one-way Anova (tukey), ns; not significant. (F). Composite profile of HIRA enrichment over 49 core ISGs. Shaded grey boxes represent gene body sequences located between transcriptional start and end sites (TSS and TES, respectively). Y-axis indicates mean HIRA enrichment (normalised to input control) per bin width. (G) Bar graph showing the median HIRA enrichment in the gene body of 49 coding non-ISGs equal in size to core ISGs (as in E). 95% confidence interval shown. ns (not significant); one-way Anova (tukey). (H) Composite profile of HIRA enrichment at the same 49 coding non-ISGs as described in G.

It is becoming evident that PML has independent roles in the regulation of intrinsic and innate immune defences during herpesvirus infection [3, 9, 32–34, 68]. To test whether PML facilitates the HIRA dependent induction of innate immune defences, we performed ChIP-seq analysis to evaluate the distribution of HIRA across the human genome in cells in which PML was depleted or not (Fig 6). Primary fibroblast cells were stably transduced with lentiviral vectors expressing PML-targeting or non-targeting control shRNAs (shPML or shCtrl, respectively; [27]). Western blot analysis confirmed PML depletion (Fig 6A). Cells were either mock treated (nt) or treated (t) with IFN-β for 24 h prior to native ChIP and NGS, as described [69]. Peak calling revealed a genome wide increase in HIRA binding to host genes following IFN stimulation in both control and PML-depleted cells (Fig 6B, S7 Table; [17]). Notably, the pattern of HIRA binding to genes was altered in IFN treated PML depleted cells in comparison to IFN treated control cells (Fig 6C, condition ii *vs* iii; *P* < 0.001). These data suggest a role for PML in the enrichment of HIRA binding to a specific subset of genes following IFN stimulation. In order to investigate this, we examined the levels of HIRA enrichment over a panel of equivalent sized interferon or non-interferon stimulated coding gene bodies (49 genes per condition; [84]). Following IFN-β treatment, the level of HIRA enrichment on ISGs was significantly reduced in PML depleted cells in comparison to IFN treated control cells (Fig 6D–6F, S7 Table; a representative example for *Mx1* is shown in Fig 6D). While a modest increase in HIRA enrichment was observed for non-interferon stimulated genes, the level of enrichment was not significant nor was it dependent on PML (Fig 6G and 6H). Collectively, these data demonstrate that PML plays a role in the enrichment of HIRA onto ISGs following IFN stimulation. Taken together with our transcriptomic analysis (Fig 5), these data demonstrate that IFN treatment stimulates HIRA binding to the gene bodies of a variety of ISGs in a PML-dependent manner to facilitate their transcriptional upregulation.

### HIRA is required for the efficient induction of ISG expression during HSV-1 infection

**Fig 7:**
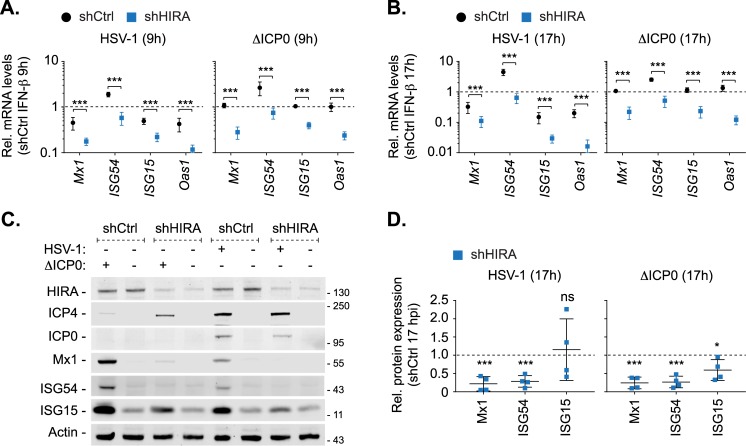
HIRA is required for efficient ISG expression during HSV-1 infection. HFt cells were stably transduced to express non-targeting control (shCtrl) or HIRA-targeting (shHIRA) shRNAs. Cells were stimulated with IFN-β or infected with 1 PFU/cell of WT or ΔICP0 HSV-1 for 9 or 17 h (as indicated). (A, B) qRT-PCR quantitation of *Mx1*, *ISG54*, *ISG15*, and *OAS1* mRNA levels in shCtrl or shHIRA cells infected with WT or ΔICP0 HSV-1 (as indicated) at 9 or 17 hpi (A and B, respectively). n = 3, means and SD shown. Values expressed relative to shCtrl + IFN-β at either 9 or 17 h (1; dotted line). *** *P* < 0.001; two-tailed t-test. (C) shCtrl or shHIRA cells were mock infected or infected with 1 PFU/cell of WT or ΔICP0 HSV-1 for 17 h. Whole cell lysates were analysed by western blot for HIRA, viral IE proteins (ICP0, ICP4), ISG (Mx1, ISG54, ISG15) expression levels, and actin (as a loading control). Molecular mass markers are shown. (D) Quantitation of ISG expression levels (as in C). n = 4, means and SD shown. Values normalized to their respective actin loading controls and expressed relative to their normalized levels in mock infected shCtrl cells (1; dotted line). * *P* < 0.05, ** *P* < 0.01, *** *P* < 0.001, ns (not significant); two-tailed t-test.

As HSV-1 infection can inhibit the termination of host gene transcription, which could influence the interpretation of our transcriptomic analysis (Fig 5; [85]), we performed validation studies to analyse the expression of a subset of ISGs (*Mx1*, *ISG54*, *ISG15*, and *OAS1*) during WT or ΔICP0 HSV-1 infection in control and HIRA depleted HFt cells. Consistent with our transcriptomic analysis, ΔICP0 HSV-1 infection of control cells efficiently induced ISG transcription to levels equivalent to those observed for IFN-β treatment alone (Fig 7A and 7B; dotted line). As expected, this host response was impaired during WT HSV-1 infection at both 9 and 17 hpi (Fig 7A and 7B; dotted line). HIRA depleted cells infected with either WT or ΔICP0 HSV-1 had a significant decrease in ISG transcript levels relative to infected control cells (Fig 7A and 7B). Western blot analysis of infected whole cell lysates demonstrated that expression of these ISGs was significantly reduced in HIRA depleted cells infected with HSV-1 (WT or ΔICP0; Fig 7C and 7D). In agreement with our qPCR analysis, the levels of ISG expression during WT HSV-1 infection of control cells was lower than that observed during ΔICP0 HSV-1 infection, consistent with ICP0 disruption of host innate immune defences [9, 36, 57, 58]. While the induction of ISG transcription was also significantly reduced in IFN-β-treated cells depleted for HIRA relative to control cells (S8A Fig; dotted line), only a minor reduction in ISG protein expression was observed (S8B and S8C Fig). These data corroborate our transcriptomic analysis (Fig 5H) and demonstrate that HIRA plays a key role in the regulation of ISG expression in response to virus infection, a host response likely to be saturated under elevated levels of exogenous cytokine stimulation. We conclude that HIRA plays a critical role in the regulation of host innate immune defences that are activated in response to HSV-1 infection under low MOI conditions (≤ 1 PFU/cell) pertinent to a clinical setting. These data identify for the first time a role for HIRA in the induction of innate defences in response to infection with replication-competent herpesvirus.

### HIRA independently contributes to the intrinsic and innate immune defences to HSV-1 infection

**Fig 8:**
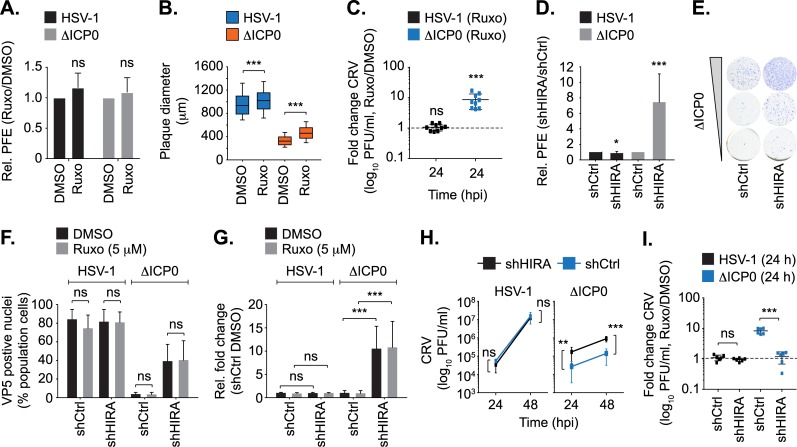
HIRA mediates intrinsic and innate immune defences to HSV-1 infection. HFt cells were DMSO or Ruxolitinib (5 μM; Ruxo) treated and infected with serial dilutions of WT or ΔICP0 HSV-1 for 24 h prior to immunostaining for plaque formation. (A) Plaque counts expressed relative to DMSO control (# plaques Ruxo / # plaques DMSO) at equivalent input dilutions and presented as relative (Rel.) plaque formation efficiency (PFE). n ≥ 3, means and SD shown. ns (not significant); two-tailed t-test. (B) Quantitation of WT or ΔICP0 HSV-1 plaque diameter within infected cell monolayers treated with DMSO or Ruxo (as in A). Boxes: 25^th^ to 75^th^ percentile range; black line: median diameter (μm); whiskers: 5^th^ to 95^th^ percentile range. n ≥ 300 plaques per sample population derived from a minimum of three independent infections. *** *P* < 0.001; Mann-Whitney *U*-test. (C) HFt cells were DMSO or Ruxo treated and infected with WT or ΔICP0 HSV-1 (0.001 or 1 PFU/cell, respectively). Cell released virus (CRV) was harvested at 24 hpi and titred (log_10_ PFU/ml) on U2OS cells. Value expressed relative to titres determined on equivalently infected DMSO-treated cell monolayers. n = 3 independent experiments conducted in triplicate, all data points are shown. Lines: median titres. *** *P* < 0.001 Mann-Whitney *U*-test. (D) shCtrl or shHIRA cells were infected with WT or ΔICP0 HSV-1 (as in A). Plaque counts are expressed relative to infected shCtrl cell monolayers at equivalent input dilutions of virus and presented as relative PFE. n≥4, means and SD shown. * *P* < 0.05, *** *P* < 0.001; two-tailed t-test. (E) Representative example of increased PFE during ΔICP0 HSV-1 infection of shHIRA cells (as described in D). (F/G) shCtrl or shHIRA cells were treated with DMSO or Ruxo (5 μM) and infected with 2 PFU/cell of WT or ΔICP0 HSV-1. Cells were fixed and permeabilized at 6 hpi prior to VP5 detection by indirect immunofluorescence and confocal microscopy. (F) Quantitation of the population of VP5 antigen positive cells as a percentage (%) of the total population of cells examined. (G) Quantitation of the population of VP5 antigen positive cells (as in F) expressed relative to equivalently infected DMSO-treated shCtrl cells. n ≥ 250 cells per sample population derived from a minimum of four independent infections. *** *P* < 0.001, ns (not significant); Mann-Whitney *U*-test. (H) shCtrl or shHIRA cells were infected with WT or ΔICP0 HSV-1 (0.0005 or 0.5 PFU/cell, respectively). CRV was collected at 24 or 48 hpi and titred on U2OS cells. n = 2 independent experiments conducted in triplicate. Means and SD shown. (I) shCtrl or shHIRA cells were infected (as in H) and treated with DMSO or Ruxo (5 μM). CRV was collected at 24 hpi and titred on U2OS cells. Values expressed relative to titres determined on equivalently infected DMSO-treated cell monolayers. n = 3 independent experiments conducted in duplicate, all data points shown. Lines: median titres. ** *P* < 0.01, *** *P* < 0.005; two-tailed t-test.

HIRA has recently been reported to act as an intrinsic antiviral regulator to HSV-1 infection through the deposition of histone H3.3 onto vDNA in the epigenetic silencing of viral gene expression [17, 18]. In light of our observations demonstrating a role for HIRA in the induction of innate immune defences to HSV-1 infection (Figs 5–7), we investigated if HIRA performs independent roles in the intrinsic and innate immune responses to HSV-1 infection, similar to those reported for PML (Fig 8; [9]). We have previously shown that pharmacological inhibition of JAK-STAT signalling by Ruxolitinib impairs the induction of ISG expression during HSV-1 infection [9]. Inactivation of innate immune defences in Ruxolitinib treated cells did not influence the relative plaque-formation efficiency (PFE) of WT or ΔICP0 HSV-1 in comparison to equivalently-infected DMSO-treated control cells (Fig 8A; [9]). Thus, inhibition of innate immune defences does not influence the probability of either WT or ΔICP0 HSV-1 to initiate lytic replication and plaque formation by 24 hpi [9]. However, plaque measurements revealed that Ruxolitinib treatment led to an increase in WT and ΔICP0 HSV-1 plaque diameter (Fig 8B; a median increase of 8 and 40%, respectively). Titration of CRV (cell-released virus) from Ruxolitinib treated cell monolayers revealed a significant increase in ΔICP0, but not WT, HSV-1 titres relative to infected DMSO-treated control cells (Fig 8C). Thus, the induction of innate immune defences restricts the rate of ΔICP0 HSV-1 propagation and spread, but not the initiation of plaque formation [9, 37]. In contrast, PFE assays in HIRA depleted cells demonstrated an 8 to 10-fold increase in ΔICP0 HSV-1 PFE relative to equivalently-infected control cells, with only a modest (< 2-fold) inhibitory effect on WT HSV-1 PFE (Fig 8D and 8E). Correspondingly, infection of HIRA depleted cells resulted in a 10-fold increase in VP5 (major capsid protein and HSV-1 late gene product) positive cells following ΔICP0, but not WT, HSV-1 infection at 6 hpi in a JAK-independent manner (Fig 8F and 8G). As the onset of vDNA replication is required to stimulate HSV-1 late gene expression, we analyzed HIRA localization to input genomes (HSV-1^EdC^ or ΔICP0^EdC^) or *de novo* synthesized vDNA pulse-labelled with EdC following the initiation of HSV-1 DNA replication (S9 Fig). HIRA was readily observed to stably colocalize with ΔICP0 vDNA at 6 hpi under both labelling conditions and to restrict the levels of ΔICP0 HSV-1 gene expression; host responses antagonized by ICP0 that correlate with the degradation of PML and dispersal of PML-NB proteins during WT HSV-1 infection (S9A–S9E Fig). These data corroborate and extend findings reported by Rai *et al*. [17], and demonstrate that HIRA contributes to the intrinsic restriction of ΔICP0 HSV-1 independently of the induction of innate immune defences. These data also demonstrate that the stable recruitment of HIRA to infecting viral genomes occurs with temporally distinct kinetics to that observed for PML and PML-NB associated restriction factors (Fig 1; [9]).

As expected, viral release assays demonstrated significantly elevated titres of CRV during ΔICP0, but not WT, HSV-1 infection of HIRA depleted cells relative to equivalently-infected control cells at 24 and 48 hpi (Fig 8H; [17]). Importantly, while Ruxolitinib treatment of infected control cells significantly enhanced ΔICP0 HSV-1 CRV titres relative to DMSO treatment, Ruxolitinib treatment of infected HIRA depleted cells failed to equivalently increase ΔICP0 HSV-1 titres (Fig 8I). These data demonstrate that HIRA plays a key role in the cytokine-mediated restriction of ΔICP0 HSV-1 propagation following the saturation of intrinsic host defences and the onset of productive virus infection. We conclude that HIRA plays independent roles in the regulation of intrinsic and innate immunity to HSV-1 by restricting the initiating cycle of viral gene expression and the induction of host innate immune defences that limit virus propagation, respectively. These combined host defences are antagonized by ICP0 during WT HSV-1 infection through the degradation of PML, which disperses HIRA from infecting viral genomes and reduces HIRA-mediated transcriptional upregulation of ISGs. We conclude that HIRA plays a crucial role in regulating multiple phases of host immunity in response to HSV-1 infection.

## Discussion

The replication-independent deposition of histone H3.3 into host chromatin has been predominantly linked to two histone chaperone complexes, namely Daxx/ATRX and HIRA/UBN1/CABIN1, which play important roles in the regulation of host chromatin structure, transcription, and DNA repair [86, 87]. The Daxx/ATRX complex typically mediates H3.3 deposition into cellular nucleosomes associated with telomeric and pericentric heterochromatin [88–92]; while the HIRA/UBN1/CABIN1 complex has been linked to the deposition of H3.3 into more transcriptionally accessible euchromatin and at sites of DNA damage [89, 93–96]. Recent reports have shown the HIRA H3.3 chaperone complex localizes at PML-NBs in response to infection with replication-defective herpesviruses to promote the assembly of viral genomes into chromatin for subsequent epigenetic silencing [17, 18]. However, the kinetics of HIRA recruitment to infecting viral genomes and PML-NBs under lytic replication conditions had yet to be examined. Here we report that HIRA displays spatiotemporally distinct kinetics of recruitment to infecting HSV-1 genomes and PML-NBs, which independently contribute to the regulation of intrinsic and innate immune defences in response to herpesvirus infection.

We recently reported that core PML-NB associated proteins (PML, Sp100, Daxx, and ATRX) rapidly (< 90 mpi, post-addition of virus) associate with and entrap infecting herpesvirus genomes as a component of the intrinsic antiviral immune response [9]. With respect to Daxx/ATRX, these findings are consistent with their reported roles as intrinsic host factors that contribute to the entrapment, chromatin assembly, and silencing of viral gene expression at PML-NBs independently of the induction of innate immune defences [9, 18, 19, 29–31]. In contrast to the stable recruitment of PML to infecting viral genomes (Fig 1A and 1B; [9]), we did not observe the stable enrichment of HIRA to either vDNA or PML-NBs at 90 or 180 mpi (Fig 1A–1C). As bio-orthogonal nucleic acid labelling is not compatible with live-cell kinetic studies, we cannot rule out the possibility of highly transient or low antigen abundance HIRA interactions with vDNA or PML-NBs (Fig 1A). Nevertheless, our observations are in contrast with the stable recruitment phenotypes reported for HIRA at PML-NBs in response to infection with replication-defective herpesviruses at 2–6 days post-infection ([17, 18]; discussed further below) or IFN stimulation 8–24 hours post-treatment (Fig 3D; [17]). Under productive infection conditions, HIRA recruitment to infecting viral genomes could only be observed following the saturation of PML-NB host defences and the onset of ΔICP0 HSV-1 DNA replication by 6 hpi (S9 Fig; [9]). These data demonstrate that stable recruitment of HIRA to vDNA occurs with temporally distinct kinetics to that of core PML-NB host factors (Fig 1; [9]), and provides supporting evidence that ΔICP0 HSV-1 genomes can be restricted at multiple phases of infection to influence the progression of viral gene expression (Fig 8D–8G, S9D and S9E Fig; [9, 19, 31]). This HIRA-mediated host response is antagonised by ICP0, in a manner analogous to, but temporally distinct from, that of Daxx/ATRX [9, 29]. ICP0 disrupts the stable localization of HIRA at vDNA without inducing its proteasome-dependent degradation (Fig 1E, S9A–S9C Fig). While the mechanism(s) of this disruption remains to be fully established, the degradation of SUMO-conjugated proteins by the STUbL-like properties of ICP0 is known to inhibit the recruitment of Daxx/ATRX to infecting viral genomes and early-stage vDNA replication complexes [9, 22, 23, 97]. Thus, ICP0 is likely to inhibit the localization of HIRA at vDNA indirectly through the degradation of other substrates that mediate HIRA recruitment or retention at vDNA independently of the degradation of HIRA itself. While this correlates with the ICP0-dependent degradation of PML (S9 Fig), a known interaction partner of HIRA [82], further investigation is warranted to test the underlying mechanism as many chromatin-associated host factors are asynchronously recruited to vDNA during the initial stages of HSV-1 replication [10, 98–100]. Our data supports and corroborates conclusions drawn by Rai *et al*., which identified HIRA to play an important role in the intrinsic antiviral restriction of ΔICP0 HSV-1 [17], and emphasizes the importance of multiple histone chaperone complexes (Daxx/ATRX and HIRA/UBN1/CABIN1) in the spatiotemporal regulation of intrinsic immune defences during different phases of herpesvirus infection [29, 31, 79]. While HIRA depletion did not significantly influence the levels of WT HSV-1 replication under low MOI conditions (0.0005 PFU/cell; Fig 8D and 8H), we could observe diminished levels of WT HSV-1 gene expression at early time points post-infection (2–6 hpi, S9D Fig). We therefore cannot discount a transient role for HIRA in the formation of viral chromatin that may promote the onset of viral gene expression [8, 53]. Nor can we discount a repressive role for HIRA at PML-NBs in other infection contexts, for example during the establishment of viral latency in sensory neurones, where the presence or absence of specific viral or cellular factors may influence the mechanism of viral chromatin assembly and silencing [18]. It is likely, therefore, that the role(s) of HIRA in the regulation of viral chromatin assembly that can lead to viral genome silencing will be dependent on the context of infection (presence or absence of ICP0), permissiveness of cell-type to restrict ΔICP0 HSV-1 replication, and stage of host immune response to infection (see below).

Following the productive onset of lytic replication, HIRA stably colocalized with PML-NBs in ICP4 negative cells at the periphery of developing ΔICP0 HSV-1 plaques. HIRA recruitment to PML-NBs under these infection conditions occurred in a JAK-dependent manner and was significantly impaired during WT HSV-1 infection (Fig 2). These data are consistent with an established role for ICP0 to disrupt PRR activation and the induction of innate immune signalling cascades that lead to IFN-β production and secretion [9, 12, 36, 45, 101]. We therefore identify that virus-induced cytokine signalling cascades to play an important role in the spatiotemporal localization of HIRA at PML-NBs during infection of normal diploid cells. Replication-defective herpesviruses promote the activation of PRRs that induce innate immune defences and cytokine secretion [33, 55, 56, 59, 102], which likely accounts for the stable localization of HIRA at PML-NBs observed under replication-defective infection conditions [17, 18]. These data are consistent with findings that IFN stimulation alone is sufficient to induce the re-localization of HIRA to PML-NBs in a range of primary and immortalized cell-types (Fig 3G; [17]) in a PML- and Sp100-dependent manner (Fig 4). Further investigation is warranted to determine why this recruitment is impaired in carcinoma cell-types that are responsive to IFN signalling (Fig 3G, S5 Fig), but may reflect changes in PML-NB composition, CDK expression levels, or host chromatin epigenetic status, which are frequently altered upon carcinoma transformation. Collectively, our data demonstrate that HIRA recruitment to PML-NBs in the context of lytic replication in diploid cells represents a sequential step in the paracrine activation of innate immune defences to prime cells for imminent infection. These data have important implications with respect to the interpretation of previous host factor recruitment studies that have utilized ΔICP0 HSV-1 (or similar) plaque-edge recruitment assays [21, 103, 104], as these recruitment phenotypes should now be considered in the context of an activated innate immune response. Correspondingly, further experiments are warranted in order to establish if the localization of HIRA at PML-NBs following replication-defective herpesvirus infection is a direct consequence of vDNA entry into the nucleus alone or an indirect consequence of PRR-induced cytokine-mediated autocrine signalling.

Under replication-competent infection conditions, our transcriptomic and infection biology analysis clearly demonstrate that HIRA plays important roles in the induction of innate immune defences and ISG expression during HSV-1 infection that correlates with its enrichment of localization at PML-NBs (Figs 2, 5 and 7). HIRA depletion abrogated the cytokine-mediated restriction of ΔICP0 HSV-1 (Fig 8I), a viral mutant known to be hypersensitive to IFN [50–52]. Thus, we identify for the first time a requirement for HIRA in the induction of innate immune defences that contribute to the cellular restriction of virus propagation, host defences impaired by ICP0 during the initiating cycle of WT HSV-1 infection (see above). While HIRA has been reported to be enriched across the human genome in response to cytokine stimulation [17], including a subset of ISGs [17, 60, 61], here we demonstrate that PML promotes the enrichment of HIRA onto ISG bodies (Fig 6, S7 Table). These data are consistent with a growing body of evidence to support a role for PML and PML-NBs in the regulation of innate immune defences in response to virus infection [9, 32–34, 68, 83]. HIRA has been reported to promote histone H3.3 deposition into nucleosomes associated with ISGs to facilitate RNA pol. II transcript elongation [60, 61]. As Daxx maintains a soluble non-nucleosomal pool of histone H3.3 at PML-NBs [17, 18, 82, 88, 105], the HIRA chaperone complex may transit through PML-NBs in response to IFN stimulation to load histone H3.3 for ISG nucleosome deposition. Thus, PML depletion may impair HIRA enrichment onto ISG bodies by dispersing the concentrated free pool of non-nucleosomal histone H3.3 at PML-NBs [82, 88]. Notably, PML-NBs are also known to localize in close proximity to transcriptionally-active gene-dense chromosome regions and to influence chromatin looping [106–109], including regions associated with the MHC locus [107]. The localization of HIRA at PML-NBs may therefore concentrate HIRA to regions of host chromatin that are transcriptionally activated in response to IFN-stimulation to enhance histone H3.3 deposition to promote their rapid expression, a hypothesis consistent with the HIRA-dependent upregulation of MHC class I genes observed in our study (Fig 5G; [107]). Alternatively, PML may enhance HIRA binding to ISGs by stimulating the onset of transcription directly through the loading of STAT-1/-2 and HDAC-1/-2 onto ISG promoters [33, 110]. Importantly, none of these hypothesises are mutually exclusive and further studies are required to identify the role(s) of PML and PML-NBs in the HIRA-dependent induction of ISG expression during herpesvirus infection.

Surprisingly, while both transcriptomic and qPCR studies demonstrate a role for HIRA in the transcriptional upregulation of ISGs in response to exogenous IFN-β stimulation (Fig 5H, S8A Fig), depletion of HIRA only had a minor impact on ISG protein expression (S8B and S8C Fig). These data imply that HIRA mediated induction of ISG expression is saturable under elevated levels of cytokine stimulation (≥ 100 IU/ml). In the context of virus infection, these data suggest that HIRA is likely to play a critical role in the regulation of innate immune defences under physiological infection conditions that result in comparatively low levels of cytokine secretion, for example in the context of WT herpesvirus infections that express a full complement of immune antagonists (Figs 5H and 7). This observation is likely to have an important role *in vivo*, where tissue physiology will further limit cytokine diffusion prior to immune cell migration and the priming of adaptive immune responses [111, 112].

In summary, we show that the histone H3.3 chaperone HIRA plays independent roles in the regulation of intrinsic and innate immune defences to HSV-1 infection that restrict the initiation and propagation of herpesviruses, respectively. We identify these immune defences to occur at spatiotemporally distinct phases of infection, highlighting a critical role for HIRA in the regulation of multiple phases of host immunity in response to pathogen invasion.

## Materials and methods

### Cells and drugs

Primary human foreskin fibroblast (HFs) cells were obtained from Thomas Stamminger (University of Erlangen; [27]). Primary human lung fibroblast (MRC5 and IMR-90) cells were obtained from the ATCC (CCL-171 and CCL-186, respectively). HFs and MRC5 cells were immortalized by retrovirus transduction to express the catalytic subunit of human telomerase (HFt and MRC5t, respectively), as previously described [25]. Primary and hTERT immortalized human fibroblasts, human retinal pigmented epithelial (RPE-1; ATCC, CRL-4000), human keratinocyte (HaCat; AddexBio, T0020001), human osteosarcoma (U2OS and SAOS; ECACC, 92022711 and 89050205), human cervical carcinoma (HeLa; a gift from R. Everett, MRC-UoG CVR), human lung adenocarcinoma (A549; a gift from B. Hale, University of Zurich), and human embryonic kidney (HEK 293T; a gift from R. Everett, MRC-UoG CVR) cells were grown in Dulbecco’s Modified Eagle Medium (DMEM; Life Technologies, 41966). HFt and MRC5t cells were cultured in the presence of 5 μg/ml of Hygromycin (Invitrogen, 10687–010) to maintain hTERT expression. Transduced HFt and IMR-90 cells expressing shRNAs were cultured in the presence of Puromycin (Sigma-Aldrich, P8833; 1 μg/ml or 0.5 μg/ml for selection or maintenance, respectively). Medium for all cell lines was supplemented with 10% foetal bovine serum (FBS; Life Technologies, 10270), with the exception of IMR-90 cells that were supplemented with 20% FBS, plus 100 units/ml penicillin and 100 μg/ml streptomycin (Life Technologies, 15140–122). Cell lines were maintained at 37°C in 5% CO_2_. 5-Ethynyl-2’-deoxycytidine (EdC; Sigma-Aldrich, T511307), MG132 (Calbiochem; 474790), Flavopiridol (CDK inhibitor; Selleckchem, S1230), CHIR-99021 HCl (GSK-3α/β inhibitor; Selleckchem, S2924), and Ruxolitinib (JAK inhibitor; Selleckchem, S1378) were prepared in DMSO and used at the indicated concentrations. Interferon beta (IFN-β; Calbiochem, 407318) was prepared in Milli-Q H_2_O and used at the indicated concentrations.

### Viruses

Wild-type (WT) HSV-1 strain 17syn+ (HSV-1), its ICP0-null mutant derivative *dl*1403 (ΔICP0; [47]), and their respective variants that express eYFP.ICP4 [113] were propagated in RPE cells and titrated in U2OS cells, as described [49]. The purification of EdC labelled HSV-1 virions was performed as essentially described [9]. Briefly, RPE cells were infected with WT or ΔICP0 HSV-1 (MOI 0.001 or 0.5 PFU/cell, respectively). At 24 h post-infection (hpi), EdC was added at a final concentration of 1 μM every 24 h until extensive cytopathic effect was observed. Supernatants containing labelled cell-released virus (CRV) were clarified by centrifugation (423 x*g* for 10 min), filtered through a 0.45 μm sterile filter, and purified through NAP-25 Sephadex column (GE Healthcare; 17-0852-01) prior to titration in U2OS cells [49].

### Plasmids and lentiviral transduction

Plasmids encoding short hairpin (sh) RNAs against a non-targeted control sequence (shCtrl; 5’-TTATCGCGCATATCACGCG-3’), PML (shPML; 5’-AGATGCAGCTGTATCCAAG-3’), ATRX (shATRX; 5’- CGACAGAAACTAACCCTGTAA-3’), Daxx (shDaxx; 5’-GGAGTTGGATCTCTCAGAA-3’), Ubc9 (shUbc9; 5’- GAAGTTTGCGCCCTCATAA-3’), Sp100 (shSp100; 5’-GTGAGCCTGTGATCAATAAT-3’), or HIRA (shHIRA clone F2; 5'-TGAATACCGACTTCGAGAAAT-3', clone F3; 5'-TCAGGACCGTTAGCCATAATC-3', clone F4; 5’-TGAATACCGACTTCGAGAAAT-3’) were used to generate lentiviral supernatant stocks for transduction of HFt or IMR-90 cells as described [23, 27–29, 79]. Pooled stably transduced cells were used for experimentation.

### Antibodies

The following antibodies were used for immunofluorescence or western blotting: Primary rabbit polyclonal: anti-actin (Sigma-Aldrich, A5060), anti-Daxx (Upstate, 07–471), anti-ATRX (Santa Cruz, H300), anti-PML (Bethyl Laboratories, A301-167A; Jena Biosciences, ABD-030), anti-Sp100 (GeneTex, GTX131569), anti-Mx1 (Santa Cruz, sc-50509; ProteinTech, 13750-1-AP), anti-ISG15 (ProteinTech, 15981-1-AP), anti-ISG54 (IFIT2, proteinTech, 12604-1-AP), and anti-histone H3 (abcam, ab1791). Primary mouse monoclonal: anti-HIRA (Millipore, 04–1488), anti-ICP0 (11060, [114]), anti-ICP4 (58s, [115]), anti-VP5 (DM165, [116]) anti-UL42 (Z1F11; [117]), and anti-PML (abcam, ab96051). Primary antibodies were detected using the following secondary antibodies: DyLight-680 or -800 conjugated goat anti-rabbit or -mouse (Thermo; 35568 and SA5-35571), Alexa -488, -555, or -647 conjugated donkey anti-rabbit or -mouse (Invitrogen; A21206, A21202, A31572, A31570, A31573, A31571), or HRP conjugated goat anti-mouse (Sigma-Aldrich, A4416).

### Plaque forming efficiency (PFE) assays

Unless otherwise stated, cells were infected with serial dilutions of HSV-1 or ΔICP0 and rocked every 10 min for 1 h prior to overlay with medium supplemented with 2% Human Serum (HS; MP Biomedicals, 2931149). 24 to 36 hpi, cells were washed twice in PBS (Sigma-Aldrich, D1408), simultaneously fixed and permeabilized in 1.8% formaldehyde (Sigma-Aldrich, F8775) and 0.5% NP40 (BDH, 56009) in PBS for 10 min, then washed twice in 0.1% Tween in PBS (PBST). Cells were blocked with 5% skimmed milk powder (SMP; Marvel) in PBST (blocking buffer) for 30 min before incubation with an anti-VP5 monoclonal antibody diluted in blocking buffer for 90 min. Cells were washed three times with PBST, incubated with HRP conjugated anti-mouse IgG diluted in blocking buffer for 60 min, then washed with PBST three times. Plaques were visualized with True Blue peroxidase developing solution (Insight, 50-78-02) according to the manufacturer’s instructions, and washed with Milli-Q H_2_O prior to plaque counting or imaging using an Axio Observer Z.1 microscope (Zeiss) with differential interference contrast. For plaque formation efficiency (PFE) assays, plaque counts are expressed relative to the number of plaques on infected control cell monolayers at the equivalent dilution of input virus. Results are presented as relative fold change (number of plaques sample/number of plaques control). Plaque diameters were measured using Zen blue (Zeiss) imaging software.

### Viral yield assays

Cells were pretreated with 5 μM Ruxolitinib or DMSO as a carrier control for 1 h prior to infection with HSV-1 (MOI 0.001 or 0.0005 PFU/cell, as indicated), or ΔICP0 (MOI 1 or 0.5 PFU/cell, as indicated). Following absorption, cell monolayers were washed twice with DMEM prior to overlay with medium containing 5 μM Ruxolitinib or DMSO. Supernatants containing CRV were collected at the indicated times post-infection. Virus titres were calculated by titration on U2OS cells, as described [49].

### Immunoprecipitation assays

3x10^6^ cells were treated with IFN-β (100 IU/ml) for 24 h prior to harvesting by trypsinization and sedimentation by low speed centrifugation (1500 rpm for 5 minutes at RT). Cell pellets were resuspended in 2 ml of ice-cold IP buffer (50 mM HEPES pH 7.4, 1% NP40, 150 mM NaCl, 10% glycerol, 1mM EDTA, 2 mM Dithiothreitol) containing a cocktail of protease inhibitors (Roche, 11873 580 001). Cell suspensions were cup horn sonicated at 4°C (Branson digital sonifier 450; 2x 30 second pulses at 20% amplitude) prior to clarification by ultra-centrifugation at 25,000 rpm for 20 mins (Beckman OptimaMax-XP ultra). Cell lysates were pre-cleared using 30 μl of equilibrated Protein G beads (Millipore, 16–201) at 4°C for 30 mins with rotation. Beads were collected by centrifugation (1 min at 13,000 rpm) and the clarified lysate transferred to a fresh tube. 1 μg of purified rabbit IgG (Sigma-Aldrich, I5006) or rabbit anti-Sp100 (GeneTex, GTX131569) was incubated with 1 ml of clarified lysate at 4°C for 2 h with rotation. Immune complexes were captured by adding 30 μl of equilibrated Protein G beads at 4°C for 90 mins. Beads were collected by centrifugation (1 min at 13,000 rpm) and washed 3x in 500 μl of IP buffer prior to final resuspension in 1x SDS-PAGE loading buffer (see below). Samples were boiled for 10 mins prior to SDS-PAGE and western blot analysis.

### Western blot

Treated or infected cells were washed twice with PBS. Whole cell lysates were collected in 1x SDS-PAGE loading buffer containing 2.5 M Urea (Sigma-Aldrich, U0631) and 150 mM Dithiothreitol (DTT; Sigma-Aldrich, D0632). Proteins were resolved on NuPAGE 4–12% Bis-Tris Protein gels (Invitrogen, NP0322BOX) in MES (Invitrogen; NP0002) or MOPS buffer (Invitrogen, NP0001) and transferred onto 0.2 μm nitrocellulose membrane (Amersham, 15249794) for 90 min at 30 volts in Novex transfer buffer (Invitrogen, NP0006-1) according to the manufacturer’s instructions. Membranes were blocked in PBS with 5% FBS (Block) for a minimum of 1 h at room temperature. Membranes were incubated in primary antibody diluted in Block for a minimum of 1 h, washed three times with PBST for 5 min each, then incubated in secondary antibody diluted in Block for 1 h. Following three 5 min washes in PBST, one 5 min wash in PBS, and one rinse in Milli-Q H_2_O, membranes were imaged on an Odyssey Infrared Imager (LiCor). The intensity of protein bands was quantified with Odyssey Image Studio Software.

### Immunofluorescence and confocal microscopy

Cells were seeded overnight on to 13 mm coverslips prior to treatment or infection at the indicated MOI and times at 37°C. For click chemistry assays, cells were washed in serum free DMEM prior to overlay in complete medium or fixation. At indicated times, cells were washed twice in CSK buffer (10 mM HEPES, 100 mM NaCl, 300 mM Sucrose, 3 mM MgCl_2_, 5 mM EGTA), simultaneously fixed and permeabilized in 1.8% formaldehyde and 0.5% Triton-X100 (Sigma-Aldrich, T-9284) in CSK buffer for 10 min, and washed twice in CSK. Coverslips were then blocked with 2% HS in PBS for 30 min prior to click chemistry followed by immunostaining. Where applicable, EdC-labelled vDNA was detected using the Click-iT Plus EdU Alexa Fluor 555 Imaging Kit (ThermoFisher scientific, C10638) according to the manufacturer’s instructions. For host and viral protein labelling, cells were incubated with primary antibodies diluted in 2% HS in PBS for 60 min, then washed in 2% FBS in PBS three times, before incubation with secondary antibodies and DAPI (Sigma-Aldrich, D9542) in 2% HS in PBS for 60 min. Coverslips were then washed in 2% FBS in PBS three times, and twice in Milli-Q H_2_O prior to mounting on Citiflour AF1 (Agar Scientific, R1320). Coverslips were examined using a Zeiss LSM 880 confocal microscope using the 63x Plan-Apochromat oil immersion lens (numerical aperture 1.4) using 405 nm, 488 nm, 543 nm, and 633 nm laser lines. Zen black software (Zeiss) was used for image capture, generating cut mask channels, and calculating weighted colocalization coefficients. Exported images were processed with minimal adjustment using Adobe Photoshop and assembled for presentation using Adobe Illustrator.

### Plaque-edge recruitment assays

HFt cells were infected with WT (MOI 0.001 PFU/cell) or ΔICP0 (MOI 2 PFU/cell) HSV-1 expressing EYFP.ICP4 to enable the initiation of viral replication and plaque formation to occur, as previously described [49]. At 24 hpi, infected cell monolayers were fixed and immunostained, as described above.

### Cell proliferation assay

HFt cells were seeded into 48-well plates at a density of 6x10^4^ cells/well. 24 h post-seeding, the cells were washed 2x in DMEM medium containing low serum (1% FBS) prior to incubation in low serum medium for 24 h. Cells were released from cell cycle arrest by the addition of DMEM medium (10% FBS) containing 1 μM EdC and either DMSO (carrier control) or GSK-3α/β inhibitor (CHIR-99021 HCl) at the indicated concentrations. Cells were fixed and permeabilized at the indicated time points prior to click chemistry (as described above). The number of EdC positive nuclei (proliferating cells containing *de novo* replicated DNA) were quantified using an automated Celigo imaging cytometer (Nexcelom biosciences), as per the manufacture’s instructions.

### Quantitative RT-PCR

Cells were mock, IFN-β stimulated (100 IU/ml), or infected with HSV-1 or ΔICP0 at the indicated MOI. Total RNA was isolated at the indicated times post-treatment using the RNAeasy Plus Kit (Qiagen, 74134), according to the manufacturer’s instructions. Reverse transcription (RT) was performed using the TaqMan Reverse Transcription Reagents kit (Life Technologies, N8080234) with oligo(dT) primers. cDNA samples were analyzed in triplicate using TaqMan Fast Universal PCR Master Mix (Life Technologies, 4352042) with the following TaqMan gene specific primer-(FAM/MGB) probe mixes (Life Technologies): assay ID PML (Hs00231241_m1), Ubc9 (Hs00163336_m1), Daxx (Hs00985566_g1), ATRX (Hs00997529_m1), Sp100 (Hs00162109_m1), HIRA (Hs00231498_m1), Mx1 (HS00895608_m1), ISG15 (Hs01921425_s1), ISG54 (Hs01922738_s1), OAS1 (Hs00973635_m1), or GAPDH (4333764F) on a 7500 Fast Real time PCR system (Applied Biosystems). Relative mRNA levels were determined using the ΔΔCt method (normalized to GAPDH) and expressed relative to indicated treatments. Mean (RQ) and standard deviations (RQmin/max) are presented.

### RNA sequencing (RNA-Seq)

2x10^5^ cells/well were seeded into 12-well dishes and incubated at 37°C for 24 h prior to experimentation. Cell monolayers were mock treated, stimulated with IFN-β (100 IU/ml) or infected with WT or ΔICP0 HSV-1 (MOI 1 PFU/cell) for 17 h. Total RNA from three technical replicate experiments was isolated using an RNAeasy Plus kit (Qiagen, 74134; as described above). RNA concentration and integrity was determined using Qubit Fluorimeter (Life Technologies, Q32855 and Q32854) and Agilent 4200 TapeStation (Agilent, 5067–5579 and 5067–5584) reagents and instruments, respectively. All samples had a RIN score of ≥ 8.5. 500 ng of total RNA was used to prepare libraries for sequencing using an Illumina TruSeq Stranded mRNA HT kit (Illumina, 20020594) and SuperScript2 Reverse Transcriptase (Invitrogen, 18064014) according to the manufacturer's instructions. Libraries were pooled in equimolar concentrations and sequenced using an Illumina NextSeq 500 sequencer (Illumina, FC-404-2005). At least 95% of the reads generated presented a Q score of ≥ 30. Reads were quality assessed using FastQC software (https://www.bioinformatics.babraham.ac.uk/projects/fastqc/), sequence adaptors removed using TrimGalore (https://www.bioinformatics.babraham.ac.uk/projects/trim_galore/), and sequences aligned to the human genome (GRCh38.86) via Ensembl (Ensembl genome browser 86) using HISAT2 [118]. FeatureCount [119] was used to count reads mapped to individual annotated gene files. EdgeR was used to calculate relative gene expression levels [120]. False discovery rate (FDR) values were calculated using the Benjamini–Hochberg method in EdgeR for each pairwise comparison (Q value). Sequences have been deposited in EMBL-EBI (https://www.ebi.ac.uk/ena) under accession number PRJEB27501. Pathway analysis was conducted using Reactome (https://reactome.org) [121, 122]. High-confidence Ensembl gene IDs (Q < 0.0001, ≥ log2 fold change) were converted to uniprot IDs using the Reactome mapping tool and used to identify pathways enriched (over-represented) for mapped entities (S1–S5 Tables). Reactome FDR values < 0.05 were considered significant for pathway enrichment, ranked, and plotted as heat maps in GraphPad Prism.

### Chromatin immunoprecipitation and sequencing (ChIP-Seq)

Native ChIP was performed for HIRA as described [69] using an equimolar mixture of mouse mAbs to HIRA (WC15, WC19, WC117, WC 119; [74], a gift from Professor Peter Adams (Beatson Institute for Cancer Research, University of Glasgow) or mouse mAb to HA tag (Covance, MMS-101R) as a species and class-matched negative control. Analysis of the ChIP-seq data was performed by assessing read quality using FASTQC (https://www.bioinformatics.babraham.ac.uk/projects/fastqc/) and trimming reads using Trim-Galore (https://www.bioinformatics.babraham.ac.uk/projects/trim_galore/). Single-end ChIP-seq reads were aligned to the human genome (hg19) using Bowtie2 [123]. Additional quality and filtering steps were undertaken; demarking duplicate reads with Picard Tools (http://picard.sourceforge.net) as well as by retaining reads that map uniquely to single loci (Alignment Statistics: S7 Table). Peak calling was performed using USeq (v8.6.0) [124], with window and extension sizes of 200 bp and 150 bp, respectively, utilizing non-specific input DNA as a control. Venn diagrams were drawn using the package Eulerr [125] and the significance and fold change of each overlap was determined with a bespoke script (https://github.com/neilrobertson/BICRCode) using a permutation based approach over 1000 iterations. HIRA enrichment on ISG bodies was compared against a random, equally sized subset of genes from the Ensembl gene set with status ‘known’ and biotype ‘coding’ (22,008 total genes; version 73 of the archive). The HIRA signal for any given window was calculated as the total number fractional reads within a window, with the product divided by the total number of reads in the dataset divided by one million. Composite profiles were generated by dividing gene length into 40 bins, each corresponding to 2.5% of the total gene length, with ten additional 500 bp windows extending from the transcriptional start and end sites of each gene (± 5 kb). The average normalised ChIP-seq signal was then calculated for each window and normalised to input DNA. Sequences have been deposited in GEO (https://www.ncbi.nlm.nih.gov/geo/) under accession number GSE128173.

## Supporting information

S1 Fig HIRA monoclonal antibody (mAb) validation. HFt cells were stably transduced to express non-targeting control (shCtrl), Daxx- (shDaxx; secondary control), or HIRA- targeting (shHIRA; clones F2-F4) shRNAs. (A) Western blot analysis of the expression levels of HIRA (anti-HIRA mAb 04–1488, Millipore) in whole cell lysates derived from shCtrl, shDaxx, or shHIRA cells. Actin and histone H3 are shown as loading controls. (B) qRT-PCR quantitation of *HIRA* mRNA levels in shCtrl, shDaxx, or shHIRA cells. Means (RQ) and SD (RQ min/max) shown and expressed relative to shCtrl cells. (C) Confocal microscopy images showing the nuclear localization of HIRA (anti-HIRA mAb 04–1488, Millipore; green) and PML (red) in shCtrl or shHIRA (clone F4) cells. Nuclei were stained with DAPI (blue). (D) Quantitation of the relative (Rel.) HIRA and PML signal intensity per nm^2^ in shCtrl or shHIRA (clone F4) cells (as indicated). n ≥ 250 cells from a minimum of three independent experiments. Boxes: 25^th^ to 75^th^ percentile range; black line: median signal intensity; whiskers: 5^th^ to 95^th^ percentile range. Values expressed relative to mean HIRA or PML signal intensity per replicate in shCtrl cells. *** *P* < 0.001, ns (not significant); Mann-Whitney *U*-test.
(EPS)

S2 Fig HIRA is not recruited to PML-NBs in mock infected or uninfected cells on the periphery of a developing WT HSV-1 plaque. Representative confocal microscopy images for quantitated data presented in Fig 2C. Wide-field images showing the nuclear localization of HIRA (red) and PML (cyan) in cells mock infected or in close proximity to a developing WT HSV-1 plaque-edge (eYFP.ICP4, green). Nuclei were stained with DAPI (blue). Cut mask (yellow) highlights regions of colocalization between HIRA and PML. Weighted colocalization coefficients are shown. Red arrows highlight eYFP.ICP4 positive cells.
(EPS)

S3 Fig HIRA is recruited to PML-NBs in non-productively infected cells on the periphery of a developing ICP0-null mutant HSV-1 plaque. Split channel confocal microscopy images for data presented in Fig 2A. Wide-field images showing the nuclear localization of HIRA (red) and PML (cyan) in cells in close proximity to a developing ICP0-null mutant HSV-1 plaque-edge (eYFP.ICP4, green). Nuclei were stained with DAPI (blue). Red and white arrows highlight representative examples of eYFP.ICP4 positive negative cells, respectively.
(EPS)

S4 Fig HIRA recruitment to PML-NBs in cells at the periphery of a developing ΔICP0 HSV-1 plaques is JAK-dependent. Representative confocal microscopy images for quantitated data presented in Fig 2D. Wide-field images showing the nuclear localization of HIRA (red) and PML (cyan) in cells in close proximity to a developing ΔICP0 HSV-1 plaque-edge (eYFP.ICP4, green) in the presence of Ruxolitinib (Ruxo.; 5 μM) or DMSO (carrier control). Nuclei were stained with DAPI (blue). Cut mask (yellow) highlights regions of colocalization between HIRA and PML. Weighted colocalization coefficients are shown. Red and white arrows highlight representative examples of eYFP.ICP4 positive and negative cells, respectively.
(EPS)

S5 Fig IFN-β induced HIRA localization at PML-NBs is cell-type dependent. Representative confocal microscopy images for quantitated data presented in Fig 3G. Primary (MRC5, HFs, IMR-90), immortalized (MRC5t, HFt, RPE, HaCat), or carcinoma (U2OS, SAOS, HeLa, A549) cells were (A) mock treated or (B) stimulated with IFN-β (100 IU/ml) for 24 h (as indicated). Cell monolayers were fixed and permeabilized and the nuclear localization of HIRA (green) and PML (red) were detected by indirect immunofluorescence. Nuclei were stained with DAPI (blue). Cut mask (yellow) highlights regions of colocalization between HIRA and PML. Weighted colocalization coefficients shown.
(EPS)

S6 Fig Inhibition of GSK-3α/β stimulates cell proliferation following release from cell cycle arrest. HFt cells were seeded into 48-well plates and incubated in medium containing low serum (1% FBS) for 24 h to induce cell cycle arrest. Cells were released from starvation into medium containing high serum (10% FBS), 1 μM EdC, and either DMSO (carrier control) or GSK-3α/β inhibitor (50 to 5000 nM, as indicated; GSKi, CHIR-99021 HCl). The cells were fixed and permeabilized at either 16 or 24 h post-release (as indicated) and the number of EdC positive nuclei (proliferating cells) labelled using click chemistry prior to quantitation using a Celigo imaging cytometer (Nexcelom biosciences). n = 3, means and SD shown. ns (not significant), ** *P* < 0.01, *** *P* < 0.001, **** *P* < 0.0001; one-way ANOVA (Dunnett’s).
(EPS)

S7 Fig IFN-β induced HIRA localization at PML-NBs is Sp100 dependent. (A, B) Representative confocal microscopy images for quantitated data presented in Fig 4D. HFt cells were stably transduced to express non-targeting control (shCtrl) or Sp100-targeting (shSp100) shRNAs. Cells were mock treated or stimulated with IFN-β (100 IU/ml) for 24 h (as indicated). Cell monolayers were fixed and permeabilized and the nuclear localization of HIRA (green) and Sp100 (red) were detected by indirect immunofluorescence. Nuclei were stained with DAPI (blue). Cut mask (yellow) highlights regions of colocalization between HIRA and Sp100. Weighted colocalization coefficients shown. Inset shows magnified region of interest (dashed boxes). (C) HFt cells were treated or not with IFN-β (100 IU/ml) for 24 h. Whole cell lysates (WCL) were collected and titrated amounts examined by western blot analysis to monitor HIRA expression levels. Actin is shown as a loading control. (D) HFt cells were treated with IFN-β (100 IU/ml) for 24 h prior to immuoprecipitation (IP) using rabbit polyclonal IgG or Sp100 antisera. Immunoprecipitated material was analysed by western blot for the presence of Sp100 and HIRA. Molecular mass markers are highlighted.
(EPS)

S8 Fig HIRA depletion minimally effects ISG expression following IFN-β stimulation. HFt cells were stably transduced to express non-targeting control (shCtrl) or HIRA -targeting (shHIRA) shRNAs. Cells were treated with IFN-β (100 IU/ml) for 9 or 17 h (as indicated). (A) qRT-PCR quantitation of *Mx1*, *ISG54*, *ISG15*, and *OAS1* mRNA levels in IFN-β stimulated shHIRA cells. n = 3, means and SD shown and expressed relative to shCtrl + IFN-β at either 9 or 17 h (1; dotted line). ** *P* < 0.01; *** *P* < 0.001, ns (not significant); two-tailed t-test. (B) Western blot analysis of the expression levels of ISGs (Mx1, ISG54, ISG15) and actin (as a loading control) from shCtrl or shHIRA cells stimulated with IFN-β for 17 h. (C) Quantitation of ISG expression levels in shHIRA cells (as shown in B). Values normalized to their respective actin loading controls and expressed relative to IFN-β stimulated shCtrl cells at either 9 or 17 h (1; dotted line). n ≥ 3, means and SD shown. * *P* < 0.05, ns (not significant); two-tailed t-test.
(EPS)

S9 Fig ICP0 disrupts HIRA localization to input or nascent vDNA. HFt cells were mock infected or infected with 3 PFU/cell of pre-labelled (HSV-1^EdC^ or ΔICP0^EdC^) or pulse-labelled (0.5 μM EdC upon overlay) WT or ΔICP0 HSV-1 in the presence 50 μM acycloguanasine (ACG; to enable the visualization of input pre-labelled EdC viral genomes following the onset of vDNA replication, [64]). Cells were fixed and permeabilized at 6 hpi (post-addition of virus). Infecting (pre-labelled) or *de novo* synthesized (pulse-labelled) vDNA was detected by click chemistry [9]. HIRA and PML were detected by indirect immunofluorescence. (A) Sub-nuclear localization of HIRA (green) and PML (cyan) with respect to infecting HSV-1^EdC^ or ΔICP0^EdC^ vDNA (red, white arrows) at 6 hpi. (B) Sub-cellular localization of HIRA (green) and PML (cyan) at HSV-1 or ΔICP0 vDNA replication complexes (red, white arrows) at 6 hpi. Insets show magnified regions of interest (dashed boxes). Cut mask (yellow) highlights regions of colocalization between cellular proteins of interest and vDNA (as indicated). Weighted colocalization coefficients shown. Nuclei were stained with DAPI (blue). (C) Quantitation of HIRA and PML colocalization to *de novo* replicating vDNA or between proteins of interest (as shown in B), as indicated. Boxes: 25^th^ to 75^th^ percentile range; black line: median weighted (w.) colocalization coefficient; whiskers: 5^th^ to 95^th^ percentile range; dashed line: coincident threshold (weighted colocalization coefficients < 0.2). n ≥ 40 nuclei per sample population derived from a minimum of three independent infections. *** *P* < 0.001; Mann-Whitney *U*-test. (D) shCtrl or shHIRA cells were mock infected or infected with 2 PFU/cell of WT or ΔICP0 HSV-1, as indicated. Whole cell lysates were collected at the indicated times (hpi) for western blot analysis to monitor HIRA expression levels and the accumulation of the viral immediate early (ICP0, ICP4), early (UL42), and late (VP5) gene products. Actin is shown as a loading control. Molecular mass markers are highlighted (Mkr). (E) Quantitation of the relative expression levels of UL42 and VP5 at 8 hpi in shHIRA cells. Values normalized to their respective actin loading controls and expressed relative to shCtrl at 8 hpi (1; dotted line). n = 3, means and SD shown.
(EPS)

S1 Table Pathway analysis of IFN-β stimulated or HSV-1 infected HFt shCtrl cells. Reactome (https://reactome.org/) pathway analysis of high confidence transcriptome changes (FDR Q-value ≤ 0.0001, ≥ log2 fold change) identified following IFN-β (IFNb; 100 IU/ml) or HSV-1 (WT or ΔICP0 HSV-1, annotated WT and dICP0 HSV-1; MOI 1 PFU/cell) treatment of HFt shCtrl cells. shCtrl (treatment; t)/shCtrl (no treatment; nt).
(XLSX)

S2 Table Pathway analysis of untreated HFt shCtrl and shHIRA cells. Reactome (https://reactome.org/) pathway analysis of high confidence transcriptome changes (FDR Q-value ≤ 0.0001, ≥ log2 fold change) identified between untreated (no treatment; nt) HFt shCtrl and shHIRA cells. shHIRA (nt)/shCtrl (nt).
(XLSX)

S3 Table Pathway analysis of IFN-β stimulated or HSV-1 infected HFt shCtrl and shHIRA cells. Reactome (https://reactome.org/) pathway analysis of high confidence transcriptome changes (FDR Q-value ≤ 0.0001, ≥ log2 fold change) identified following IFN-β (IFNb; 100 IU/ml) or HSV-1 (WT or ΔICP0 HSV-1, annotated WT and dICP0 HSV-1; MOI 1 PFU/cell) treatment of HFt shHIRA cells. shHIRA (t)/shCtrl (nt).
(XLSX)

S4 Table Pathway analysis of IFN-β stimulated or HSV-1 infected HFt shCtrl and shHIRA cells. Reactome (https://reactome.org/) pathway analysis of high confidence transcriptome changes (FDR Q-value ≤ 0.0001, ≥ log2 fold change) identified following IFN-β (IFNb; 100 IU/ml) or HSV-1 (WT or ΔICP0 HSV-1, annotated WT and dICP0 HSV-1; MOI 1 PFU/cell) treatment of HFt shHIRA and shCtrl cells. shHIRA (t)/shCtrl (t).
(XLSX)

S5 Table Differentially downregulated immune genes between IFN-β stimulated or HSV-1 infected HFt shCtrl and shHIRA cells. Differential downregulated immune system genes identified by Reactome (https://reactome.org/) pathway analysis of treated (t) HFt shCtrl and shHIRA cells (as described in S4 Table). shHIRA (t)/shCtrl (t).
(XLSX)

S6 Table Relative fold ISG transcriptome changes in HFt shCtrl and shHIRA cells following IFN-β stimulation or HSV-1 infection. Relative fold ISG transcriptome [84] changes (FDR Q-value < 0.05) identified following IFN-β (IFNb; 100 IU/ml) or HSV-1 infection (WT or ΔICP0 HSV-1, annotated WT HSV1 and dICP0 HSV-1; MOI 1 PFU/cell) treatment. Conditions: shHIRA (no treatment; nt)/shCtrl (nt). shCtrl (treatment; t)/ shCtrl (nt). shHIRA (t)/shCtrl (t).
(XLSX)

S7 Table HIRA ChIP-seq alignment statistic and ISG enrichment analysis. Mapped input control and HIRA ChIP-seq reads in untreated (neg.) or IFN-β treated (2000 IU/ml; Pos.) IMR-90 shCtrl and shPML cells (as indicated). Relative HIRA enrichment levels on 49 similarly sized interferon stimulated or non-interferon stimulated coding genes in mock (no treatment, [nt]) or IFN-β treated (IFNb; 2000 IU/ml) cells (as indicated).
(XLSX)
